# Survival of kidney transplantation in people living with HIV/AIDS: a systematic review and meta-analysis

**DOI:** 10.1186/s12879-025-11480-7

**Published:** 2025-09-26

**Authors:** Ka Chun Leung, Wincy Wing Sze Ng, Jonathan Ciofani, Wendy Kwok

**Affiliations:** 1 Department of Medicine and Geriatrics, Tuen Mun Hospital, Hong Kong Special Administrative Region, China; 2 Adult Intensive Care Unit, Queen Mary Hospital, Hong Kong Special Administrative Region, China; 3 Sydney Medical School, The University of Sydney, Sydney, Australia; 4 School of Public Health, Faculty of Medicine, Imperial College, London, UK

**Keywords:** Kidney Transplantation, HIV/AIDS, Human Immunodeficiency Virus, Antiretroviral Therapy, People with HIV

## Abstract

**Background:**

The 2013 HIV Organ Policy Equity (HOPE) Act expanded kidney transplantation eligibility for people with HIV/AIDS (PWH), marking a significant milestone and necessitating updated reviews on the outcomes of renal transplantations on PWH.

**Objective:**

To assess kidney transplantation outcomes in PWH, including patient and graft survival, graft rejection, and infection rates.

**Method:**

We conducted a systematic review of studies published between 1990 and 2023. Included studies reported on kidney transplantation outcomes in PWH, focusing on graft and patient survival, rejection, and infections and compared with HIV-negative kidney graft recipients.

**Result:**

Out of 743 studies, 49 prospective and retrospective studies were included, with data of 6174 PWHs analysed. At one year post-transplant, patient survival in PWH was high at 94% (95% CI: 92.83–94.97%), with a decrease to 83% beyond five years (95% CI: 76.50–87.64%). Graft survival exhibited similar trends. Graft rejection rates increased from 26% in one year to 39% over five years. There were no significant differences in short- to medium-term recipient survival rates between PWH and HIV-negative individuals.

**Conclusion:**

Kidney transplantation is a viable option for PWH, with short- to medium-term outcomes comparable to HIV-negative recipients. Future research should focus on optimizing long-term outcomes.

**Supplementary Information:**

The online version contains supplementary material available at 10.1186/s12879-025-11480-7.

## Key points

1. Kidney transplantation is a viable option for people living with HIV/AIDS.

2. Short-term patient and graft survival is comparable to HIV-negative recipients but both declines after 5 years.

3. Graft rejection for people with HIV increases over time, reaching 39% at five years post-transplant, with a consistently higher risk than in HIV-negative recipients.

4. Avoiding protease inhibitors in antiretroviral therapy may improve graft survival.

5. Hepatitis C co-infection negatively impacts transplant outcomes.

**Supplementary Information:**

The online version contains supplementary material available at 10.1186/s12879-025-11480-7.

## Introduction

As of 2022, nearly 40 million people are affected by HIV/AIDS globally, with around 1.3 million new cases annually [1]. In Europe, the incidence of end-stage kidney disease (ESKD) is 6.7 per 10,000 patient-years, which is 5 times higher than the general population [2]. The most common causes of chronic kidney disease (CKD) in people with HIV (PWH) are similar to those in the general population, including diabetes and hypertension. However, HIV-associated nephropathy (HIVAN) remains a distinct contributor, particularly among individuals of African descent due to genetic predispositions such as APOL1 gene variants [3]. Additionally, long-term exposure to certain antiretroviral therapies (ART) further exacerbates the risk [4].

ART has transformed HIV from a fatal disease into a manageable chronic condition, making organ transplantation a possible option for this population. The 2013 HIV Organ Policy Equity (HOPE) Act in the US catalyzed large-scale clinical trials in kidney transplantation for people with HIV/AIDS (PWH), marking a pivotal shift in eligibility for these patients [5]. Furthermore, the “Undetectable = Untransmittable” (U = U) concept advocated by The US Center for Disease Control and Prevention (CDC) stated that PWH who achieve and maintain an undetectable viral load through consistent adherence to ART cannot sexually transmit the virus to others [6]. This concept encouraged transplantation physicians and surgeons to consider kidney transplantation in PWH as a safe and beneficial approach, alleviating concerns of potential HIV transmission during surgical procedures or postoperative care, which evidence by increasing transplants from HIV positive donors since implementation of HOPE act [7].

Recent studies underscore the feasibility of kidney transplantation for PWH, showing comparable outcomes in graft survival, rejection, and infection rates with other ESKD patients [8–11]. Despite this progress, PWH face unique challenges, including pre-transplantation complexities, interactions between ART and immunosuppressants, and the effects of co-infections like hepatitis C on patient and graft survival. A significant gap remains in synthesized data addressing these issues [12], hindering the integration of PWH into transplantation programs globally. Furthermore, PWH face significant inequities in renal transplantation, particularly in resource-limited settings: while more than 80% PWH living in Low- and Middle-Income Countries (LMICs) [13], only 5% of them can access renal transplantation services [14].

This systematic review and meta-analysis aimed at evaluating kidney transplantation outcomes in PWH and comparing them with HIV-negative kidney transplant recipients. It examines patient and graft survival, the incidence of graft rejection and infection. The influence of different HIV-specific clinical factors (e.g. CD4 count, viral load, ART regimens) as well as the role of co-infections are evaluated. By integrating findings from both observational cohorts and clinical trials, the study seeks to provide evidence-based insights to inform clinical practice, guide policy, and promote equitable transplantation care for people living with HIV.

## Materials and methods

### Data sources and searches

Relevant bibliographic databases were searched between 1990 and 2024, including MEDLINE via Ovid, EMBASE via Ovid, MEDLINE via PubMed, and the Cochrane Central Register of Controlled Trials, covering the period from 1/1/1990 to 31/12/2024, focusing on the time period when ART was implemented in the management of PWH [15]. The search strategy utilized a combination of keywords and MeSH terms related to HIV, kidney transplantation and relevant outcomes. All searches were restricted to studies published in English. A thorough account of the search terms and strategy per database is documented in Appendix 1.

### Study outcomes

The primary outcomes of our systematic review and meta-analysis were centred on the overall recipient survival post-kidney transplantation in adults living with HIV/AIDS. The secondary outcomes included graft survival, graft rejection and infections. The impact of various potential key variables on transplantation outcomes, including antiretroviral therapy (ART), immunosuppressants, co-infections such as hepatitis C and variations in HIV viral load, were evaluated. Effect measures were reported as survival probabilities, hazard ratio, infection incidences accompanied by a 95% confidence interval. The PICO criteria was summarized in Table 1. A detailed eligibility criteria is presented in Appendix 3.

**Table 1:** Table of summarised PICO criteria

Criteria	Detail
Participants (P)	Adults with HIV/AIDS (PWH) who have undergone kidney transplantation.
Interventions (I)	Kidney transplantation
Comparators (C)	Individuals with end-stage kidney disease (ESKD) who are HIV-negative and have undergone kidney transplantation.
Outcomes (O)	Recipient and graft survival, graft rejection and post-transplant infections

### Study selection

Both randomized and non-randomized controlled trials, as well as prospective and retrospective observational have been included. Exclusion criteria include studies that are off-topic, duplicated, or contain non-relevant results. Additionally, animal studies and non-original research, such as pilot studies, reviews, letters to editors, and conference abstracts, are not considered. Titles and abstracts retrieved are screened by two independent reviewers, who apply predefined eligibility criteria to identify relevant studies(Appendix 3). Subsequently, the full texts of these potentially eligible studies are independently assessed by the same two reviewers for final inclusion in the review. Disagreements are addressed through discussion between the two reviewers. This method ensures that all included studies meet our strict criteria for relevance and quality. Detailed eligibility criteria are included in Appendix 3. Study selection and conflict resolution are completed through Covidence (https://app.covidence.org/).

### Data extraction and quality assessment

Data extraction was initially carried out by the first reviewer, with subsequent validation and agreement by the second reviewer. The extracted data from each study included the first author’s name, year of publication, study location, design methodology, sample size, outcome measures, and specific methodologies for outcome assessment. Detailed statistical estimates highlighting variables significantly correlated with the primary and secondary outcomes were also gathered. This included, where available, additional information such as specifics of the comparison group, adjustment factors, the statistical model used to derive the effect estimates, and the type of estimate used in the study, for example, Hazard Ratio (HR), Risk Ratio (RR), or Odds Ratio (OR), Survival probabilities, Incidence of outcome along with their 95% Confidence Intervals (CI).

In those studies where such details were not available, this was recorded to maintain transparency and integrity in the reporting process.

Quality assessment of the targeted studies was undertaken by Risk Of Bias In Non-randomised Studies - of Interventions (ROBINS-1) [16]. Assessments were performed separately by two reviewers. Publication bias was assessed by Egger’s Test, Begg’s rank test and funnel plot [17].

### Data synthesis and analysis

In addressing the inherent heterogeneity and diverse reporting styles across the included studies, our approach to data synthesis incorporated both meta-analytic and narrative synthesis methods. The narrative synthesis was strategically utilized to explore, interpret, and present findings from individual studies in summary evidence tables. A random-effects meta-analysis was conducted to determine outcomes suitable for quantitative synthesis (e.g. probabilities of patient survival and graft survival at definitive time points). To handle uneven distributions in the reported results, we applied mathematical transformations (e.g., converting percentages into log or logit scales) to ensure consistency during analysis.

For studies which categorized patients into HIV seropositive and seronegative groups, Hazard ratios were calculated where feasible and synthesized using the random effects model [18, 19].

For subgroup analysis, univariate and multivariate meta-regression analyses through a random-effects model were performed to synthesize and examine the impact of baseline characteristics, ART utilization, immunosuppressant regimes, and co-infections in PWH on transplantation outcomes.

Furthermore, we conducted sensitivity analyses by applying the same data synthesis process to studies categorized into different quality levels as determined in the quality assessment phase.

Statistical analyses were conducted using Python 3.11 with specific libraries including ‘pandas’, ‘scipy’, ‘statsmodels’ and ‘matplotlib’ for comprehensive and robust analysis [20–24]. A p-value of less than 0.05 was considered statistically significant across all analyses. To assess heterogeneity among the included studies, the I² statistic was utilized, with cutoff points of 25%, 50%, and 75% representing mild, moderate, and severe heterogeneity, respectively. The source code for data synthesis and creation of figures is available on GitHub (https://github.com/leungkcofficial/hiv_txp).

## Results

### Study selection and characteristics

**Fig. 1:**
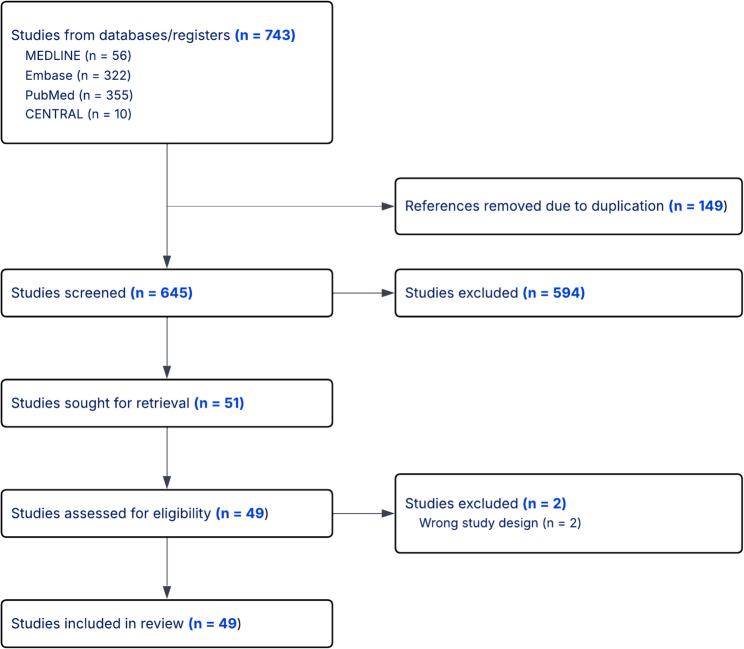
Screening and selection process

**Fig. 2:**
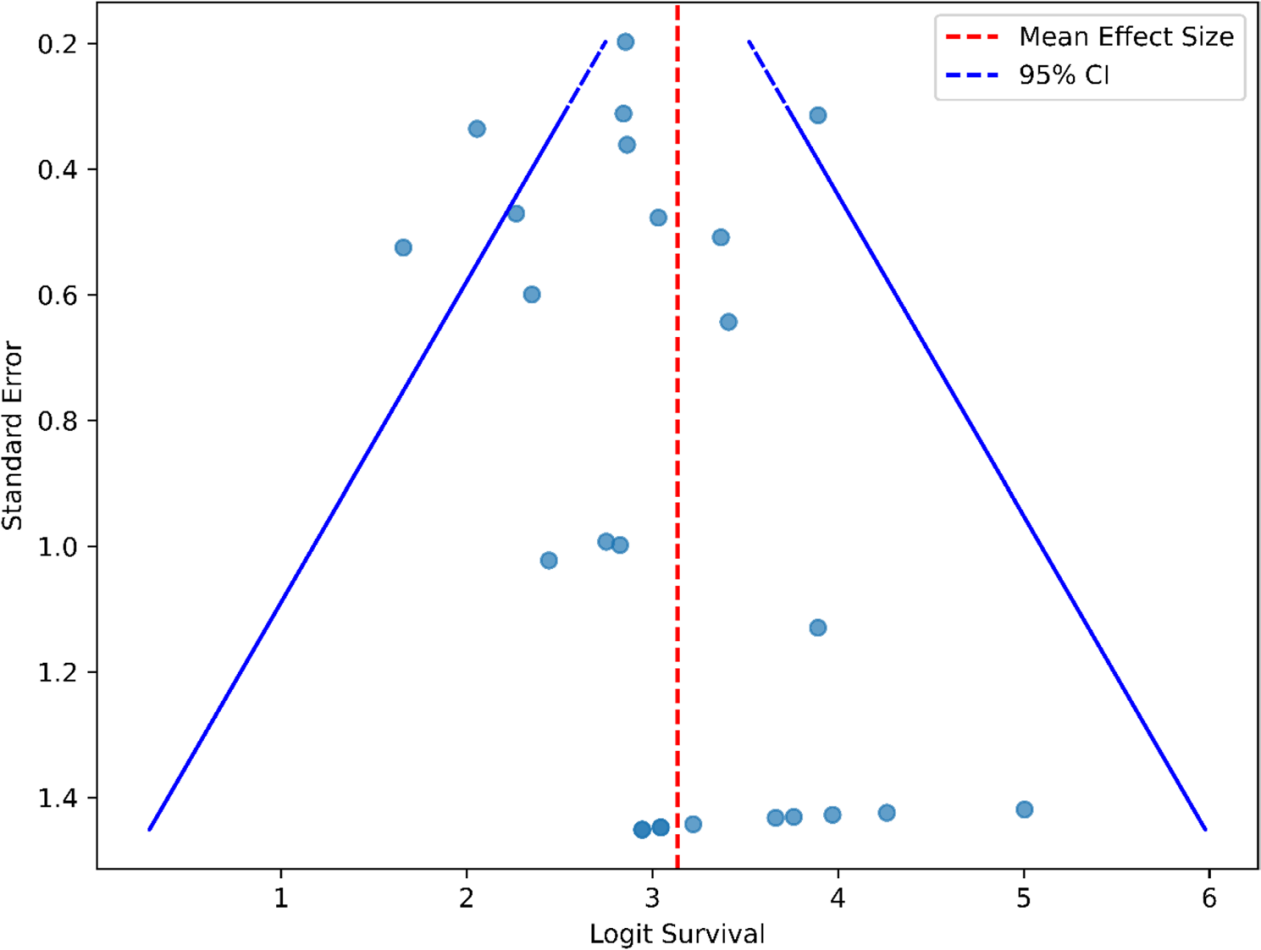
Funnel Plot for publication bias assessment

A total of 743 potentially eligible studies were identified. After removing duplicated studies and screening of titles and abstracts, 51 studies remained for full-text review. Finally, we included 49 studies for systematic review and meta-analysis. 2 studies were excluded due to non-suitable study design and result reporting [25, 26]. The included studies comprised 6 prospective and 43 retrospective observational studies, published between 1992 and 2020, originating from diverse geographical regions such as the United States, Europe, and Asia. A total of 6174 PWH kidney recipients were included in this review (Fig. 1). Of these studies, twenty reported a comparison between PWH and HIV-seronegative populations in primary outcomes [8, 27–45]. Details of the included studies are summarised in the Supplementary Table S1. While the funnel plot and Egger’s test showed asymmetry and potential publication bias, Begg’s rank test do not provide strong evidence of publication bias. The funnel plot, Egger’s test and Begg’s rank test for publication bias assessment are presented in Fig. 2; Table 2, respectively. The quality assessment result of the studies are presented in Supplementary Table S3a and S3b.

**Table 2:** Result of egger’s test and begg’s rank test

Test	Statistic	*p*-value
Egger’s test	2.448	0.015
Begg’s rank test	0.205	0.15

### Primary outcomes

#### Patient Survival (PS)

**Fig. 3:**
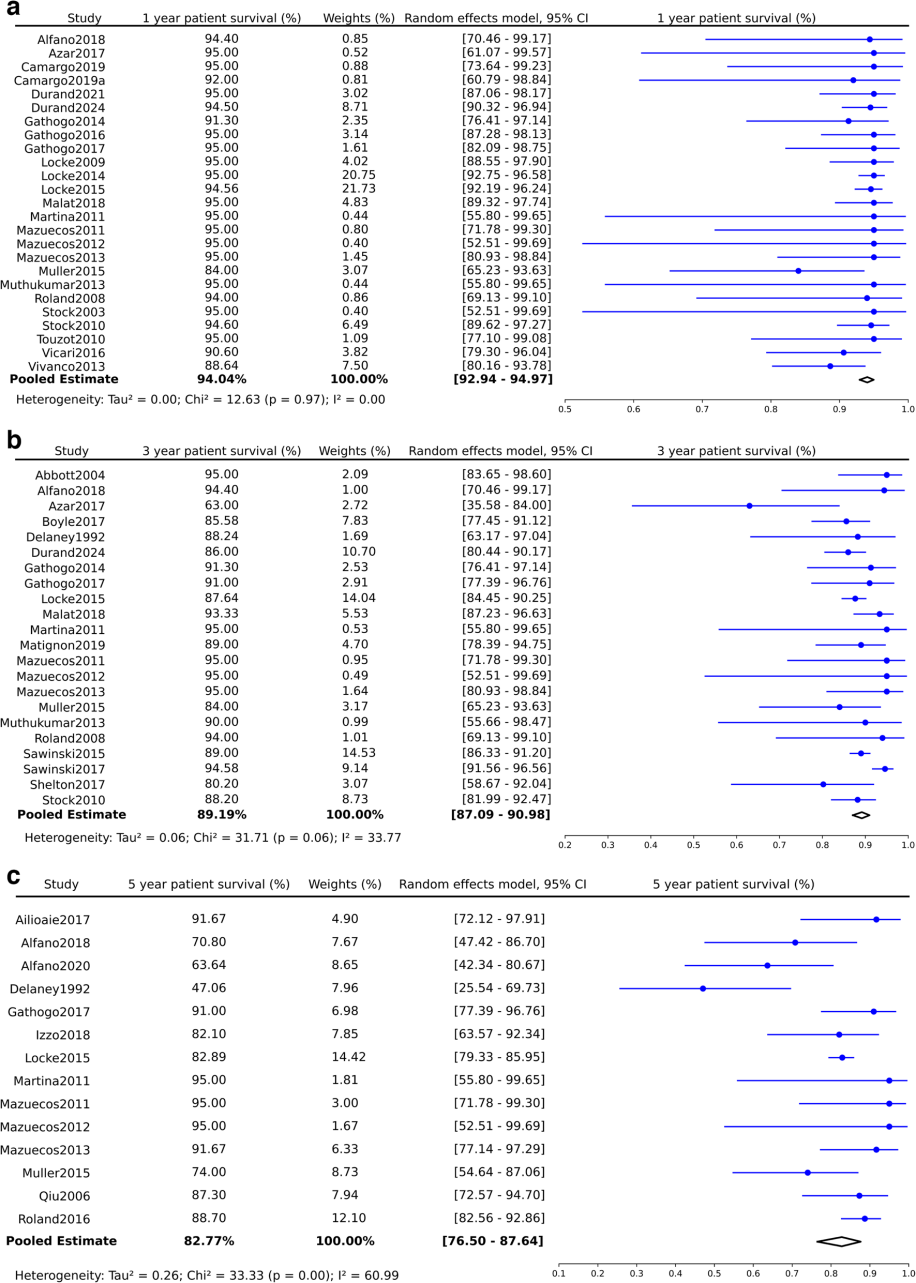
Synthesized data of patient survival in PWH kidney recipients. **a**: Patient Survival in 1 year. **b**: Patient Survival in 3 years. **c**: Patient Survival in 5 years and beyond

**Fig. 4:**
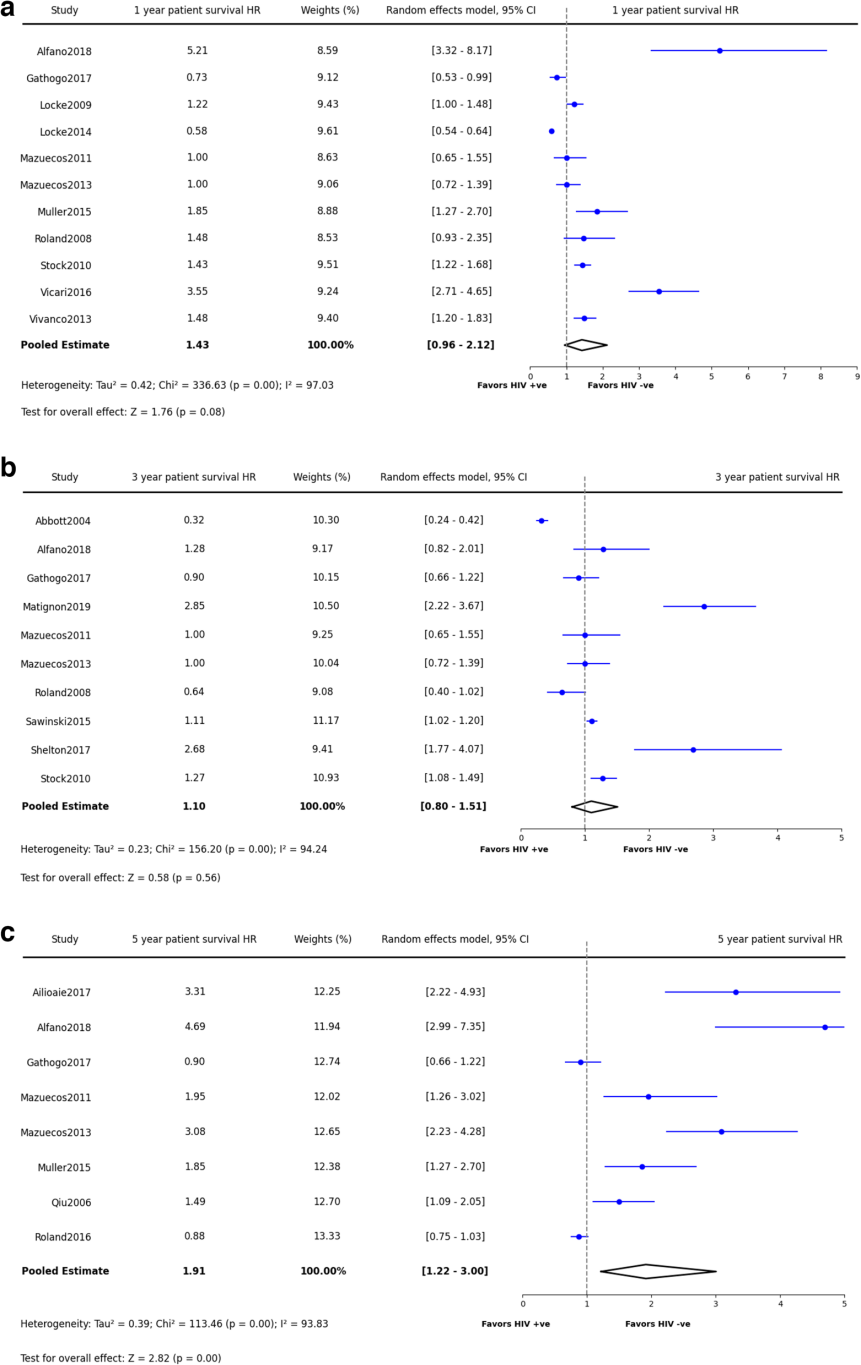
Synthesized data of patient survival hazard ratio in PWH kidney recipients. **a**: Patient Survival Hazard Ratio in 1 year. **b**: Patient Survival Hazard Ratio in 3 years. **c**: Patient Survival Hazard Ratio in 5 years and beyond

The patient survival rate at one-year post-transplantation was 94% (95% CI: 92.9–95.0%, *p* = 0.97), which slightly decreased to 89.2% at three years (95% CI: 87.1–91.0%, *p* = 0.06) and further to 82.8% for periods extending beyond five years (95% CI: 76.5–87.6%, *p* = 0.002)(Fig. 3a-c). Compared with HIV-negative populations, the 1-year and 3-year patient survival HRs revealed no significant disadvantage for PWH (1-year HR: 1.43, 95% CI: 0.96–2.12, *p* = 0.08; 3-year HR: 1.10, 95% CI: 0.80–1.51, *p* = 0.56), as depicted in Fig. 4a and b. However, a significant survival risk for PWH kidney recipients was evident after 5 years (HR: 1.91, 95% CI: 1.22–3.00, *p* = < 0.0001), as illustrated in Fig. 4c. Detailed data extraction and synthesis results are presented in Tables 3 and 4 and Supplementary Table S2.

**Table 3:** Summary of primary and secondary outcomes

Outcome Category	Time Frame	Outcome	95% Confidence Interval	I² Statistic	*P*-Value
Patient Survival (PS)	1 Year	94.0%	92.9–95.0%	0%	0.97
	3 Years	89.2%	87.1–91.0%	33.77%	0.06
	*≥* 5 Years	82.8%	76.5–87.6%	60.99%	0.002
Graft Survival (GS)	1 Year	90.5%	89.2–91.7%	0%	0.86
	3 Years	79.8%	74.6–84.1%	81.55%	< 0.0001
	*≥* 5 Years	70.5%	64.2–76.1%	50.26%	0.02
Graft Rejection (GR)	1 Year	25.1%	18.3–33.4%	89.6%	< 0.0001
	3 Years	31.0%	22.2–41.3%	72.45%	0.0002
	*≥* 5 Years	38.5%	28.3–49.8%	92.46%	< 0.0001
Post-Transplant Infection (INF)	1 Year	63.62 episodes per 100 patient-year	29.39–88.02	99.87%	< 0.0001
	*≥* 5 Years	18.2 episodes per 100 patient-years	14.09–23.18%	23.00%	0.24

**Table 4:** Comparative outcomes of kidney transplantation in PWH vs. HIV-Negative populations

Outcome Measure	Time Frame	Hazard Ratio (HR)	95% Confidence Interval	I² Statistic (%)	*P*-Value
Patient Survival	1 Year	1.42	0.96–2.12	97.03	0.08
	3 Years	1.10	0.80–1.51	94.24	0.57
	> 5 Years	1.91	1.22–3.00	95.29	0.005
Graft Survival	1 Year	2.11	1.50–2.96	95.29	< 0.0001
	3 Years	1.89	1.18–3.01	97.43	0.01
	> 5 Years	1.76	0.90–3.45	96.82	0.10
Graft Rejection	1 Year	1.88	1.45–2.44	89.83	< 0.0001
	> 5 Years	2.29	1.26–4.17	95.79	0.01

### Secondary outcomes

#### Graft survival (GS)

**Fig. 5:**
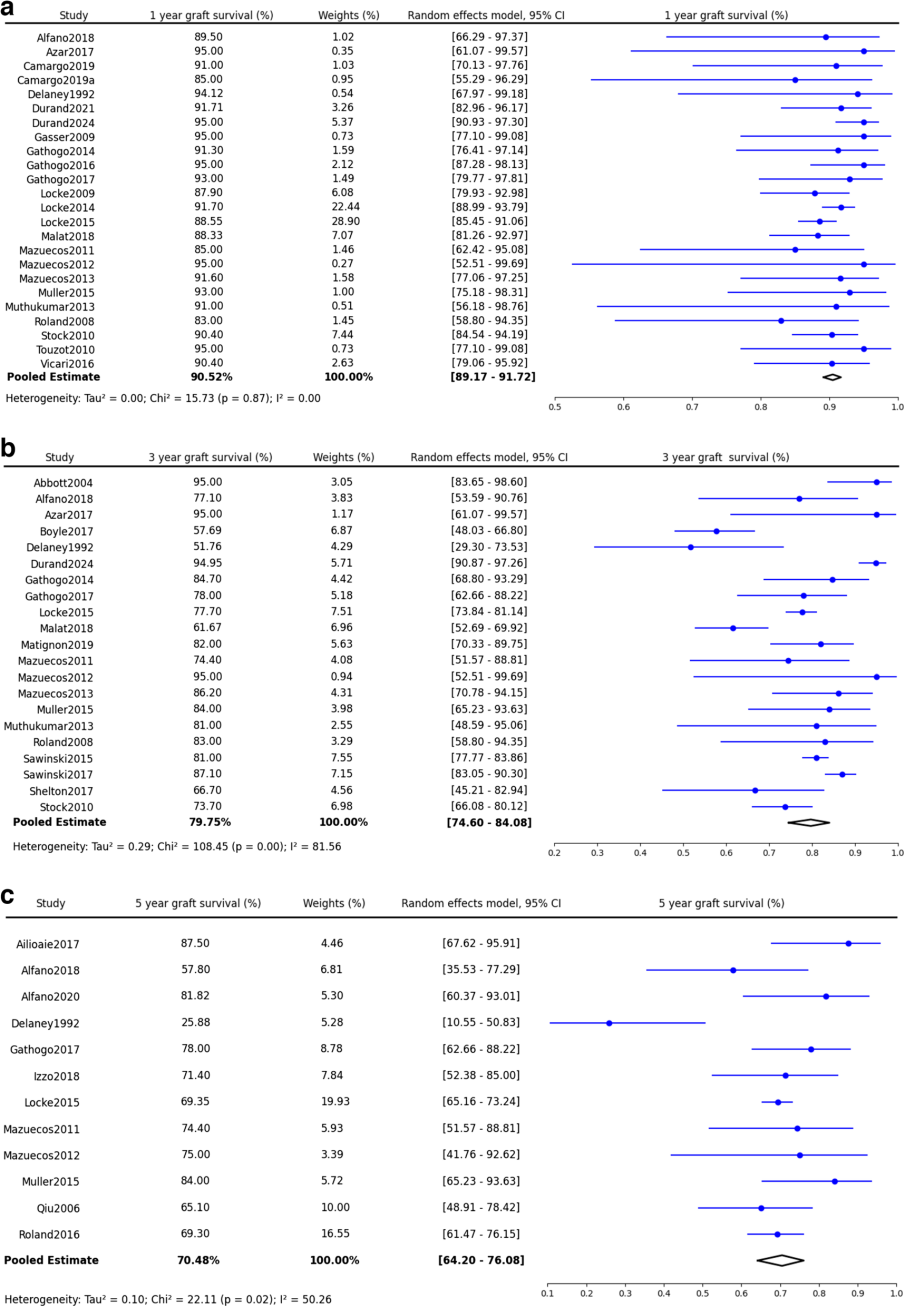
Synthesized data of graft survival in PWH kidney recipients. **a**: Graft Survival in 1 year. **b**: Graft Survival in 3 years. **c**: Graft Survival in 5 years and beyond

**Fig. 6:**
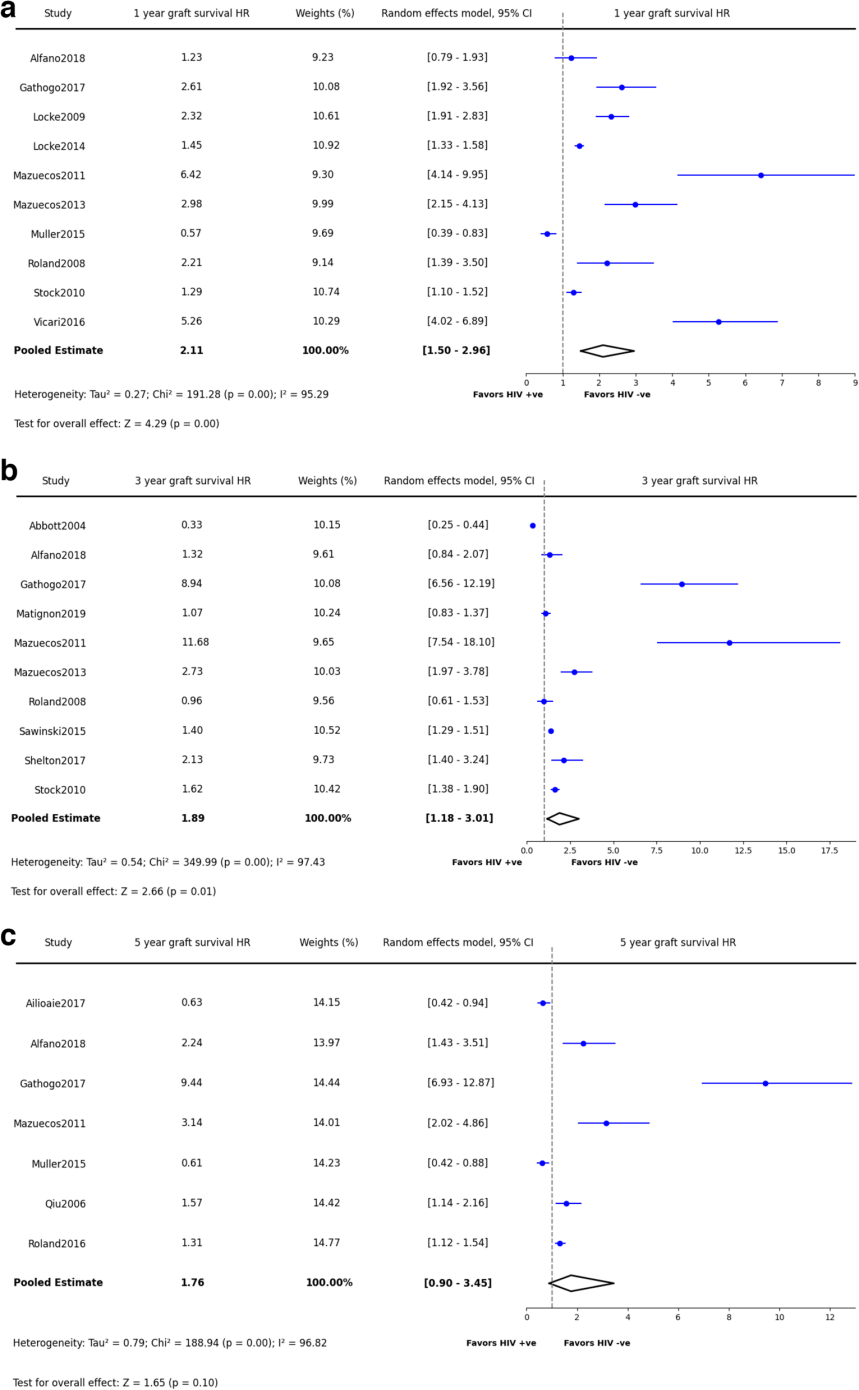
Synthesized data of graft survival hazard ratio in PWH kidney recipients. **a**: Graft Survival Hazard Ratio in 1 year. **b**: Graft Survival Hazard Ratio in 3 years. **c**: Graft Survival Hazard Ratio in 5 years and beyond

Similar to the trend of recipient survival, the one-year graft survival rate was 90% (90.2%, 95% CI: 88.8–91.5%, *p* = 0.97)(Fig. 5a) in PWH. This rate decreased over time, with a 78% survival rate at three years (77.9%, 95% CI: 72.7–82.3%, *p* < 0.0001) and further down to 71% for periods beyond five years (70.5%, 95% CI: 64.2–76.1%, *p* = 0.02)(Fig. 5a-c). Different from the recipient survival, PWH kidney recipients were found to have a statistically significant disadvantage in short and mid-term graft survival (1-year HR: 2.11, 95% CI: 1.50–2.96, p = < 0.0001; 3-year HR: 1.89, 95% CI: 1.18–3.01, *p* = 0.01)(Fig. 6a-b). There was no significant graft survival risk in PWH beyond 5 years (Fig. 6c).

#### Graft rejection (GR)

**Fig. 7:**
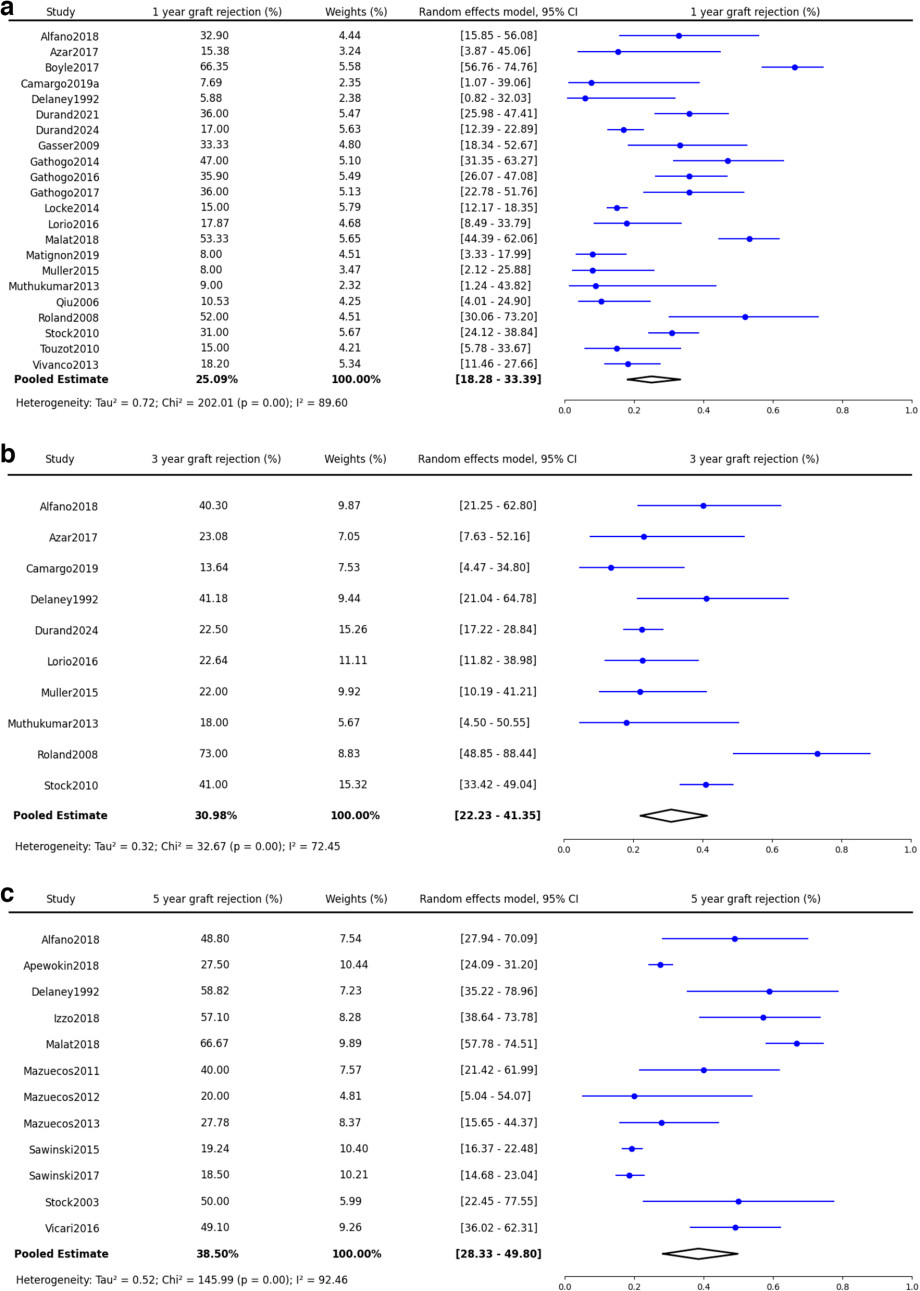
Synthesized data of graft rejection in PWH kidney recipients. **a**: Graft Rejection in 1 year. **b**: Graft Rejection in 3 years. **c**: Graft Rejection in 5 years and beyond

**Fig. 8:**
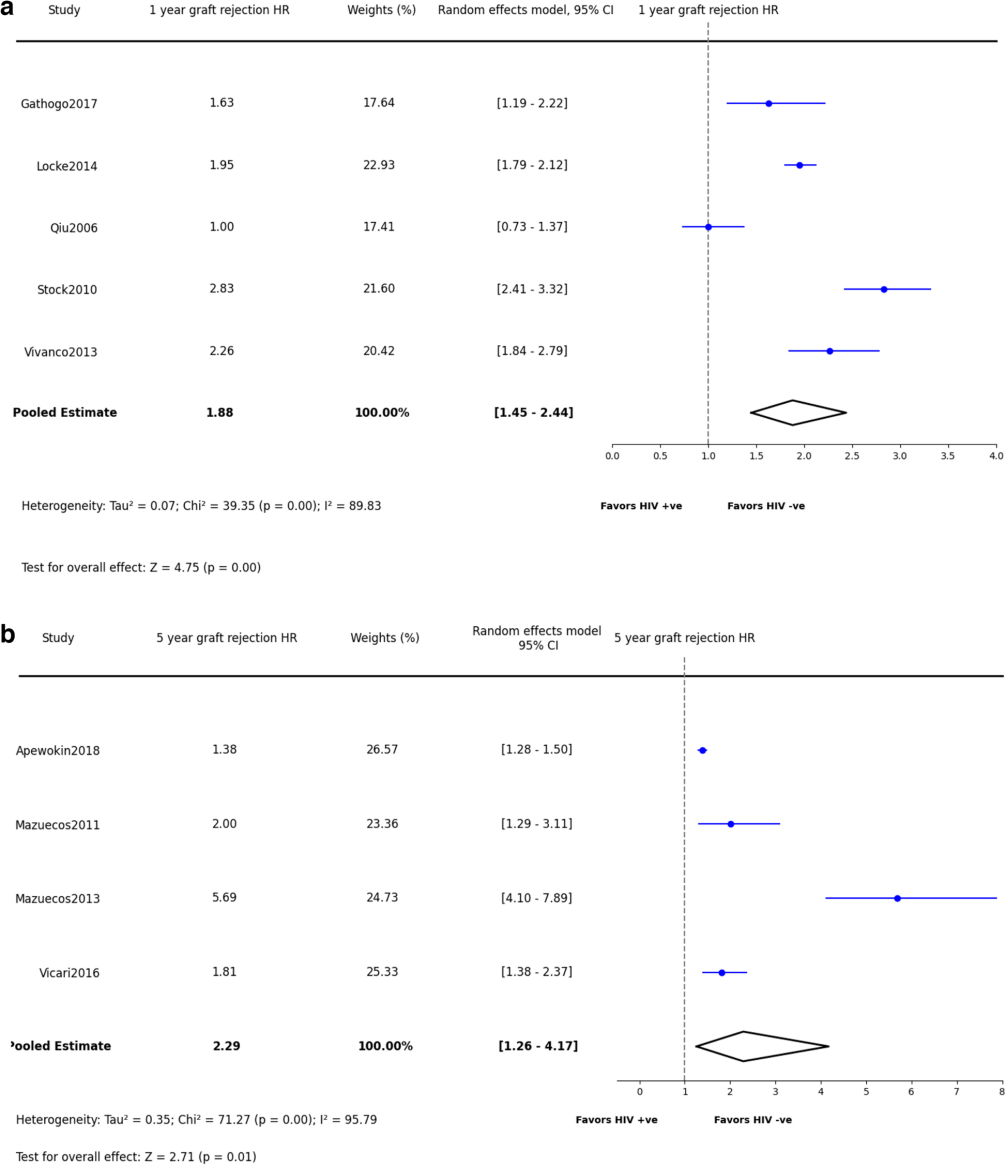
Synthesized data of graft rejection hazard ratio in PWH kidney recipients. **a**: Graft Rejection Hazard Ratio in 1 year. **b**: Graft Rejection Hazard Ratio in 5 years and beyond

Graft rejection rates were also notable; at one year, the rejection rate was 26% (25.6%, 95% CI: 18.3–34.4%, *p* < 0.0001)(Fig. 7a), increasing to 33% at three years (32.7%, 95% CI: 22.9–44.2%, *p* < 0.004)(Fig. 7b), and reaching 39% for periods over five years (38.5%, 95% CI: 28.3–49.8%, *p* < 0.0001)(Fig. 7c). Compared with HIV-negative recipients, PWH have a higher risk of graft rejection in both short and long-term (1-year HR: 1.88, 95% CI: 1.45–2.44, p = < 0.0001; 5-year HR: 2.29, 95% CI: 1.26–4.17, *p* = 0.01)(Fig. 8a and b).

#### Post-transplantation infection (INF)

**Fig. 9:**
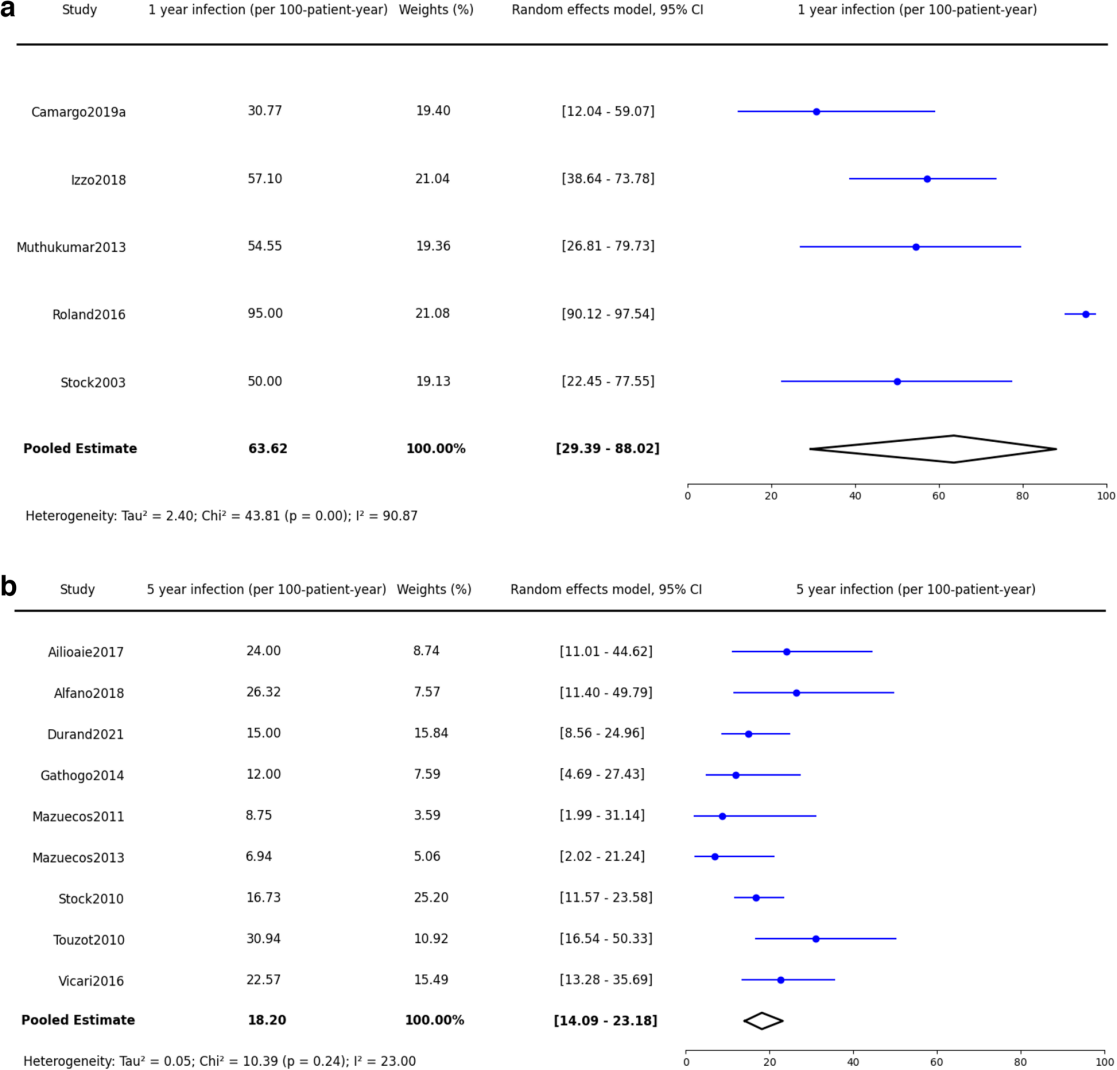
Synthesized data of post transplantation infection incidence in PWH kidney recipients. **a**: Post transplantation infection incidence in 1 year. **b**: Post transplantation infection incidence in 5 years and beyond

Furthermore, the incidence of post-transplantation infections presented significant findings. In the first year following transplantation, there were nearly 64 episodes per 100 patient-years (63.62, 95% CI: 29.39–88.02, I^2^ = 99.84%, *p* < 0.0001)(Fig. 9a). For periods extending beyond five years, the incidence reduced to 18 episodes per 100 patient-years (18.2, 95% CI: 14.09–23.18%, *p* < 0.24)(Fig. 9b).

### Subgroup analysis

A series of pre-transplant and post-transplant features of PWH kidney recipients were subjected to meta-regression analysis. While the significance of each covariable is doubtful in most univariate analyses due to a lack of enough studies providing data [46], our multivariate meta-regression identified several noteworthy associations. Avoiding protease inhibitor-based ART regimens was linked to better graft survival outcomes, and hepatitis C co-infection showed a statistically significant negative impact on both short- and long-term graft survival. (Table 5).

**Table 5:** Results of multivariate Meta-regression with random effects model

Outcome Measure	Time Frame	Moderator	Pooled Estimate	95% Confidence Interval	*P*-Value	Log-Likelihood	Adjusted *R*^2^ Statistic (%)	Number of studies recruited
Patient Survival	1 Year	Mean pre-transplantation CD4 count	0.004	0.002–0.006	< 0.0001	−7.2695	89.6	7
	3 Years	Using induction therapy during kidney transplantation	−7.97	−18.88–2.93	0.152	−4.9870	65.6	5
Graft Survival	1 Year	Hepatitis C Co-infection	−0.92	−1.64 -- −0.21	0.012	−4.7854	33.5	12
		Avoid using protease inhibitor-containing ART	0.61	0.20–1.03	0.004			
	> 5 Years	Hepatitis C Co-infection	−1.09	−1.83–0.36	0.004	−3.9432	30.2	10
Graft Rejection	1 Year	Fail to maintain HIV viral suppression	0.79	0.01–1.41	0.046	−10.1035	19.8	9

Notably, failure to maintain HIV viral suppression post-transplant was associated with increased risk of 1-year graft rejection (pooled estimate: 0.79; 95% CI: 0.01–1.41; *p* = 0.046). However, this finding should be interpreted with caution due to its borderline significance, modest explanatory power (adjusted R²: 19.8%), and relatively weak log-likelihood (Table 5).

To evaluate the impact of temporal changes in HIV and transplant care, studies were stratified into three eras: pre-1996 (before ART received FDA approval and became widely available) [47, 48], 1996–2007 (wide adoption of calcineurin inhibitor and mycophenolate mofetil in post-transplant immunosuppressant regime) and post-2007 (once daily tacrolimus received European approval, and the first CCR5 antagonist and integrase inhibitor received FDA approval) [49–52]. Only 1 study recruited recipients before 1996 [53], while 10 studies involving data between 1996 and 2007, representing 13.3% of the total PWH cohort [8, 29, 31, 32, 34, 37, 42, 43, 54, 55]. No significant difference in the primary outcome between studies before or after 2007 (Supplementary Table S4).

Additionally, 31 studies reported data from the United States [8, 29, 30, 34, 36, 37, 39, 40, 42, 43, 45, 53, 55–72], while 14 reported outcomes from Europe [11, 27, 28, 31, 32, 38, 41, 54, 73–77]. Again, no significant difference in the primary outcome was found between US or European cohorts (Supplementary Table S5), suggesting that geographic variation in practice patterns did not significantly influence the pooled effect estimate.

Detailed results of univariate meta-regression is enclosed in supplementary table S6 in the appendix.

### Sensitivity analysis

When examining patient survival, studies yielded similar survival estimates, especially in the short term no matter when they are classified according to their nature or their risk of bias. For instance, the one-year patient survival rate was 93.6% in prospective studies and 94.1% in retrospective studies, with a decreasing trend to 74.0% and 83.5% to the 5-year mark, with no significant difference compared with the main data synthesis (Table 6). Similar trend was found when the studies are stratified by their risk of bias. For example, the one-year patient survival rate was 94.9% in studies with low risk of bias, 93.3% in those with moderate risk of bias, and 95.0% in those with serious risk of bias. At three years, the rates decreased to 89.6%, 86.3%, and 92.5%, respectively, while beyond five years, survival was 86.0%, 80.3%, and 75.2% (Table 7). These results align closely with the main data synthesis.

**Table 6:** Sensitivity analysis of primary and secondary outcomes according to the nature of the studies included

Outcome Measure	Time Frame	Prospective trials			Retrospective trials		
		Survival/Rate (%)	I^2^ (%)	*P*-Value	Survival/Rate (%)	I^2^ (%)	*P*-Value
Patient Survival	1 Year	93.6	12.98	0.33	94.1	0.00	0.99
	3 Years	87.0	0.00	0.85	90.0	41.16	0.04
	> 5 Years	74.0	0.00	N/A^1^	83.5	62.28	0.002
Graft Survival	1 Year	93.6	58.75	0.06	90.0	0.00	0.94
	3 Years	85.5	88.47	< 0.0001	77.8	79.87	< 0.0001
	> 5 Years	84.0	0.00	N/A^1^	69.5	48.98	0.03
Graft Rejection	1 Year	19.8	84.46	< 0.0001	26.9	90.65	< 0.0001
	3 Years	28.7	86.17	0.0007	32.3	66.55	0.0064
	> 5 Years	50.0	0.00	N/A^2^	37.8	93.04	< 0.0001

1. Only one prospective study reported patient survival and graft survival after 5 years [33]

2. Only one prospective study reported graft rejection after 5 years [71]

**Table 7:** Sensitivity analysis of primary and secondary outcomes according to the risk of bias

Outcome Measure	Time Frame	Low risk of bias			Moderate risk of bias			Serious risk of bias		
		Survival/Rate (%)	I^2^ (%)	P-Value	Survival/Rate (%)	I^2^ (%)	P-Value	Survival/Rate (%)	I^2^ (%)	P-Value
Patient Survival	1 Year	94.9	36.74	0.08	93.3	0.00	0.79	95	0.00	1.00
	3 Years	89.6	49.99	0.02	86.3	37.40	0.16	92.5	0.00	0.79
	> 5 Years	86.0	37.02	0.15	80.3	50.91	0.08	75.2	75.03	0.04
Graft Survival	1 Year	90.9	34.29	0.12	90.9	0.00	0.83	93.3	0.00	0.92
	3 Years	79.4	87.81	< 0.0001	76.5	0.00	0.41	84.4	78.18	0.003
	> 5 Years	69.3	1.51	0.41	78.2	0.00	0.69	49.3	81.84	0.02
Graft Rejection	1 Year	27.4	93.90	< 0.0001	21.8	46.85	0.09	7.3	0.00	0.76
	3 Years	25.3	31.48	0.23	24.5	25.34	0.26	31.5	36.16	0.21
	> 5 Years	35.1	94.41	< 0.0001	58.8	0.00	N/A^2^	45.2	43.34	0.17

The sensitivity analysis echoed the main results regarding graft survival and rejection. The one-year graft survival rate remained high across both study designs, with 93.6% in prospective studies and 90.0% in retrospective ones, reflecting no significant deviation (*p* = 0.06 and *p* = 0.94)(Table 6). The prospective studies also reported a lower rejection rate (19.8%) compared to retrospective studies (26.9%) at one-year post-transplantation (Table 6).

The sensitivity analysis of the hazard ratio was also consistent with the main data synthesis. While studies with a serious risk of bias did not report the outcome of renal transplantation in HIV-negative cohorts, which precluding direct comparison, low and moderate-risk studies showed no increased risk of patient survival in 1-year and 3 years post-transplant(Table 9). Beyond five years, low-risk studies reported a substantially higher HR of 3.03 (95% CI: 1.94–4.77, *p* < 0.0001), suggesting a significant survival disadvantage in PWH, whereas moderate-risk studies showed no significant difference (HR 1.28, *p* = 0.50).

For graft survival, low-risk studies reported an HR of 2.72 (95% CI: 1.66–4.46, *p* = 0.0001) at one year, 3.47 (95% CI: 1.10–10.99, *p* = 0.03) at three years, and 2.66 (95% CI: 1.91–3.70, *p* < 0.0001) beyond five years. Moderate-risk studies reported HRs of 1.43 (95% CI: 0.81–2.53, *p* = 0.21) at one year, 2.42 (95% CI: 0.71–8.21, *p* = 0.15) at three years, and 2.39 (95% CI: 0.16–35.35, *p* = 0.52) beyond five years. Serious-risk studies did not provide HRs for graft survival due to missing HIV-negative cohort data (Table 9).

The one-year analysis indicated consistently increased rejection risks across all study types and risk of bias for graft rejection(Tables 8 and 9). This suggests that, irrespective of study quality, PWH had a higher risk of graft rejection compared to HIV-negative populations.

**Table 8:** Sensitivity analysis of hazard ratio (HR) according to the nature of the studies included

Outcome Measure	Time Frame	Prospective trials				Retrospective trials			
		HR	95% CI	Z	*P*-Value	HR	95% CI	Z	*P*-Value
Patient Survival	1 Year	1.53	1.23–1.91	3.8	0.22	1.39	0.86–2.23	1.35	0.17
	3 Years	1.27	1.08–1.49	2.95	0.003	0.94	0.78–1.13	−0.61	0.54
	> 5 Years	1.85	1.27–2.70	3.2	0.001	2.22	1.06–4.63	2.14	0.03
Graft Survival	1 Year	0.87	0.39–1.96	−0.33	0.74	2.64	1.77–3.92	4.79	< 0.0001
	3 Years	1.62	1.38–1.90	5.88	< 0.0001	3.27	1.29–8.21	2.52	0.01
	> 5 Years	0.61	0.42–0.88	−2.6	0.009	4.09	1.62–10.33	2.99	0.002
Graft Rejection	1 Year	2.83	2.41 − 3.32	12.73	< 0.0001	1.69	1.29 − 2.20	3.81	0.0001

**Table 9:** Sensitivity analysis of hazard ratio (HR) according to the risk of bias

Outcome Measure	Time Frame	Low risk of bias				Moderate risk of bias			
		HR	95% CI	Z	*P*-Value	HR	95% CI	Z	*P*-Value
Patient Survival	1 Year	1.51	0.89–2.56	1.52	0.12	1.29	0.88–1.88	1.3	0.19
	3 Years	0.96	0.74–1.24	−0.31	0.76	0.94	0.64–1.38	−0.30	0.76
	> 5 Years	3.03	1.94–4.77	4.83	< 0.0001	1.28	0.63–2.6	0.67	0.50
Graft Survival	1 Year	2.72	1.66–4.46	3.96	0.0001	1.43	0.81–2.53	1.24	0.21
	3 Years	3.47	1.1–10.99	2.12	0.03	2.42	0.71–8.21	1.42	0.15
	> 5 Years	2.66	1.91–3.70	5.83	< 0.0001	2.39	0.16–35.35	0.64	0.52
Graft Rejection	1 Year	1.69	1.20–2.38	3.03	0.0023	2.18	1.27–3.74	2.82	0.01

*All study with serious risk bias did not report the renal transplantation outcome of HIV negative patient cohort, hazard ratio cannot be calculated

### Heterogeneity assessment

To assess heterogeneity across settings, we also stratified outcomes by country income level [78]. The majority of included studies were conducted in high-income countries, while only three originated from low- and middle-income countries (LMICs) [33, 35, 79]. Due to the small number of LMIC-based studies, statistical power was limited, and no definitive conclusions could be drawn from this subgroup. Nevertheless, the available LMIC data showed no substantial deviation in short-term survival outcomes from high-income settings.

To address the significant heterogeneity observed during data synthesis, we employed a ‘leave-one-out’ approach and the Baujat plot to identify studies contributing most to the heterogeneity in each outcome [80, 81]. Three studies were identified as significant contributors to heterogeneity across outcomes [27, 30, 35]. Further analysis of study methodologies and participants’ baseline characteristics revealed several potential sources of heterogeneity, including a higher co-infection rate of cytomegalovirus [35], the use of older-generation immunosuppressants [37, 53], differences in antiretroviral therapies (e.g., CCR5 antagonists) [27], the use of national transplant registry data as a comparison cohort [30], and an imbalanced distribution of recipient ethnicity [28, 30, 35, 37, 53, 57]. The Baujat plot of outcomes with reports on heterogeneity contribution are reported on supplementary table S4 and supplementary figure S1.

## Discussion

This systematic review and meta-analysis provide a comprehensive evaluation of kidney transplantation outcomes in PWH. The findings demonstrate that, with optimal management, both patient survival and graft survival in PWH are comparable to those in HIV-negative recipients in the short to medium term following transplantation. These results corroborate recent evidence highlighting the viability of transplantation in this population when managed under structured ART regimens [82]. Both patient and graft survival show a notable decline beyond five years post-transplantation. This could be attributed to multiple factors, including a lack of long-term outcome studies and the prevalence of comorbidities such as hypertension and diabetes, which may act as confounders. Additionally, the rising rejection rates over time may lead to more intensive immunosuppressive therapy, which increases the risk of infection and contributes to patient and graft loss. Although this meta-analysis cannot evaluate causality or sequential mechanisms, the pattern of data supports the need to explore this hypothesis in future research.

Our analysis revealed a comparable risk profile for long-term graft survival and rejection rates between PWH and HIV-negative patients (Table 4). In addition to the primary findings, our finding offers significant insights into the role of specific ART regimens in kidney transplantation outcomes for PWH. Importantly, the meta-regression analysis identified that avoiding protease inhibitor (PI)-based ART regimens significantly improved graft survival at one year (Table 5). Although the transition to integrase inhibitor-based regimens did not reach statistical significance, their potential benefits, such as a reduced interaction profile and lower nephrotoxicity, warrant further investigation in the post-transplant setting. Protease inhibitors have been associated with potential drug interactions and renal graft loss, suggesting that integrase inhibitors might offer a safer and more effective alternative [70]. This aligns with the U = U advocacy, emphasizing the importance of optimal ART management to maintain viral suppression and improve transplant outcomes [6].

The finding of the potentially significant negative impact of hepatitis C co-infection is consistent with the findings in HIV-negative recipients [83–85]. However, its diminished significance in univariate analyses on graft rejection and recipient survival underscores the necessity of exploring confounding variables and integrating HCV management into long-term care strategies for PWH undergoing transplantation.

A notable concern raised in our review is the impact of induction therapy on patient survival. Our subgroup analysis suggests potential risks associated with certain induction therapies, which may affect recipient survival (Table 5). This result, potentially influenced by the varying induction therapies used in different studies and the increased risk of infection, underscores the necessity for detailed, prospective clinical trials to unravel the long-term effects of HIV and ART on organ function and the immune system, deepening our understanding of transplantation implications in this population.

There are several limitations in this review. Due to the vulnerability of PWH with chronic kidney disease (CKD), these individuals are often subject to thorough assessments and selection criteria before being included in transplantation-related studies. This selective recruitment may introduce both selection and publication bias, potentially explaining the observed graft survival benefits for PWH in prospective studies and studies with a high risk of bias (Tables 8 and 9).

Another significant limitation is the predominance of retrospective observational studies with relatively small patient populations. Given the ethical considerations surrounding patient autonomy, it is impossible to blind HIV status from both donors and recipients in clinical trials. The involvement of English-only studies in this meta-analysis may also introduce bias to the synthesized results [86]. Furthermore, although study selection and full-text screening were performed independently by two reviewers with consensus reached through discussion, the absence of a third reviewer to resolve potential conflicts may represent a limitation. In future reviews, involving a third independent reviewer could further enhance the objectivity of the study inclusion process.

For studies comparing outcomes between PWH and HIV-negative kidney recipients, the majority used data from common local registries including United States Renal Data System, Scientific Registry of Transplant Recipients and United Network for Organ Sharing rather than from the specific centers conducting the studies [8, 28–31, 36, 37, 39, 40, 42, 43, 45, 55, 65–67, 70]. Such comparisons may introduce bias due to missing data in renal registries, participant duplications due to overlapping cohorts, and the use of different transplantation protocols across centers.

Variations in ART, induction therapy, and immunosuppressant regimens contribute to considerable heterogeneity in the results, potentially impacting their generalizability. Additionally, the evolving nature of HIV care and the long-term impact of newer ART regimens on transplant outcomes remain under-researched areas. Consequently, most included studies focus on short to medium-term outcomes, leading to a gap in understanding the long-term implications for PWH undergoing kidney transplantation.

Another critical limitation is the method of data synthesis. While employing logit transformation to reduce skewness in primary outcome data is beneficial, it poses challenges in the quantitative interpretation of pooled estimates. The non-linear nature of this transformation complicates the direct comprehension of these results, potentially affecting their clinical application. Recognizing these limitations is vital for contextualizing our findings and guiding future research directions in this field. In addition, the statistical methods used for time-to-event analysis vary across included studies. While 20 studies employed Cox proportional hazards models, 14 presented Kaplan-Meier analysis with log-rank testing, and 15 did not specify the modeling strategy. The methodological differences may influence the interpretation of effect estimates and introduce additional heterogeneity and uncertainty in the pooled analysis.

One notable limitation of this study is the underrepresentation of data from low- and middle-income countries (LMICs), where over 80% of PWH reside [13]. Despite an initial objective to assess the impact of LMIC settings on kidney transplantation outcomes in PWH, only three studies included in this meta-analysis were conducted in LMICs. This gap highlights the urgent need for more robust and regionally diverse data to ensure that future findings are globally applicable and address equity issues in transplantation care.

Based on the findings of this review, several policy and intervention recommendations can be identified to improve the outcomes of kidney transplantation in PWH. First, promoting and implementing a standardised post-transplant monitoring and care protocols PWH is necessary [87]. These protocols should include regular monitoring of ART adherence and potential drug interactions, particularly when protease inhibitors are used, to minimise the risk renal graft loss. HCV co-infection screening and management program should also be implemented for PWH undergoing kidney transplantation, as hepatitis C has been identified as a significant negative factor in graft and patient outcomes.

Future research should concentrate on the long-term outcomes of kidney transplantation in PWH. Studies are needed to explore the impact of newer ART regimens on transplant success and to develop strategies for managing the increased risk of long-term complications. Prospective studies are necessary to clarify the causality or the temporal order of events such as rejection, immunosuppression, and infection. Additionally, multi-centred clinical trials in diverse demographic settings are necessary to provide a more global perspective.

## Conclusion

This systematic review substantiates kidney transplantation as a viable and effective option for PWH, showing comparable outcomes to the general population in various aspects. The findings underscore the importance of long-term management strategies, particularly in mitigating the survival disadvantages observed beyond five years. Tailored post-transplant monitoring and care protocols are crucial to optimize outcomes for this population. Future research should prioritize investigating the long-term effects of evolving ART regimens, particularly integrase inhibitor-based therapies, and their potential to improve graft and patient outcomes.

## Supplementary Information

Supplementary Material 1.

Supplementary Material 2.

## Data Availability

All data generated or analyzed during this study are included in this published article/as supplementary information files.Data extracted and all the Python code used in data analysis and synthesis can be found at https://github.com/leungkcofficial/hiv_txp.

## Abbreviations

AIDS: Acquired Immunodeficiency Syndrome
APOL1: Apolipoprotein L1
ART: Antiretroviral Therapy
CDC: Centers for Disease Control and Prevention
CCR5: C–C Chemokine Receptor Type 5
CI: Confidence Interval
CKD: Chronic Kidney Disease
ESKD: End–Stage Kidney Disease
FDA: Food and Drug Administration
GR: Graft Rejection
GS: Graft Survival
HIV: Human Immunodeficiency Virus
HIVAN: HIV–Associated Nephropathy
HOPE Act: HIV Organ Policy Equity Act
HR: Hazard Ratio
INF: Infection
KM: Kaplan–Meier
LMICs: Low–and Middle–Income Countries
OR: Odds Ratio
PICO: Participants, Interventions, Comparators, Outcomes
PS: Patient Survival
PWH: People with HIV/AIDS
ROBINS: I–Risk Of Bias In Non–randomised Studies–of Interventions
RR: Risk Ratio
U = U: Undetectable = Untransmittable
US: United States
