# Supplementary material for: Survival of kidney transplantation in people living with HIV/AIDS: a systematic review and meta-analysis
Source: BMC Infect Dis. 2025 Sep 26;25:1160. doi: 10.1186/s12879-025-11480-7 (PMC12465869; doi:10.1186/s12879-025-11480-7)
Supplement: Supplementary file 1 — Supplementary Material 1. [file 12879_2025_11480_MOESM1_ESM.docx]

### Title

Survival of kidney transplantation in people living with HIV/AIDS: A systematic review and meta-analysis.

#### Authors and affiliations

Ka Chun Leung

Resident Specialist

Department of Medicine and Geriatrics

Tuen Mun Hospital

Hong Kong Special Administrative Region

China

Wincy Wing Sze Ng

Associate Consultant

Adult Intensive Care Unit

Queen Mary Hospital

Hong Kong Special Administrative Region

China

Jonathan Ciofani

Clinical Associate Lecturer

Sydney Medical School

The University of Sydney

Wendy Kwok

Senior Teaching Fellow

School of Public Health

Faculty of Medicine

Imperial College London

#### Corresponding author

Ka Chun Leung

E-mail: kc.leung4@ha.org.hk

### Appendix 1: Supplementary Data

**Supplementary Tables:**

##### Supplementary Table S1. Summary of included studies

| Study ID | Country of study | Study type | HIV positive samples | HIV negative samples | Study period | Data source | Intervention | Outcome | Conclusion | Survival analysis strategy |
| --- | --- | --- | --- | --- | --- | --- | --- | --- | --- | --- |
| Abbott2004(1) | USA | Retrospective cohort study | 47 | 27804 | 1/1/1996 – 31/10/2001 | United States Renal Data System | retrospective cohort analysis of U.S. adult deceased donor kidney transplant recipients between 1996 and 2001, focusing on the impact of HIV infection on mortality and graft loss. | HIV-infected recipients had improved survival compared with HIV-uninfected recipients, though this was not statistically significant in adjusted analysis. It also noted that HIV-infected kidney transplant recipients were less likely to be African American. | Kidney transplantation in HIV-infected patients is plausible and ongoing but highlighted that HIV-infected candidates who underwent kidney transplantation were demographically unrepresentative of HIV-infected candidates generally. | CoxPH |
| Ailioaie2017(2) | France | Retrospective case-control study | 24 | 21 | 1/2004 – 12/2015 | Single center | retrospectively analyzed data from 24 HIV-infected kidney transplant recipients matched to 21 non-infected controls. It aimed to evaluate the outcomes of HIV-infected kidney transplant recipients, particularly focusing on infectious complications. | Similar incidence of post-transplant infections in HIV-infected renal transplant recipients compared to non-infected controls. It also noted that infections, but not HIV status, had adverse effects on patient and graft survival. | With current antiretroviral therapy and immunosuppressive protocols, the rate of infectious complications and patient and kidney graft survival are similar between HIV-infected kidney transplant recipients and HIV-negative controls. However, post-transplant infections appear to have a more significant impact on outcomes than HIV status. | KM |
| Alfano2018(3) | Italy | Retrospective Cohort Study | 19 | 200 | 3/2008 – 9/2017 | Single center | Kidney transplantation in HIV+ patients | Patient and graft survival, incidence of acute rejection, HIV control | Kidney transplantation was safe and effective in HIV+ recipients. One- and three-year graft and patient survival rates were comparable to HIV- controls, though five-year patient survival was significantly lower. High prevalence of metabolic and endocrine complications post-transplantation was noted. | KM |
| Alfano2020(4) | Italy | Retrospective cohort study | 22 | 0 | 2008 - 2019 | Single center | Comparison of Maraviroc-treated group (10 patients) vs. Maraviroc-free group (12 patients) in HIV-infected kidney transplant recipients | Half of the Maraviroc-treated patients developed graft rejections (7 episodes, mostly T-cell mediated)  Five episodes of graft rejection in the Maraviroc-free group  No statistically significant difference in graft rejection rates between the two groups (P = .23) | Use of Maraviroc was ineffective in preventing graft rejections in this cohort of patients. | n/a |
| Apewokin2018(5) | USA | Nationwide analysis using the Inpatient Databases of the Healthcare Cost & Utilization Project spanning 2008-2013 | 605 | 103532 |  | Healthcare Cost & Utilization Project | The analysis compared clinical and health care utilization outcomes between HIV-positive and HIV-negative recipients. | The study found that clinical and fiscal outcomes are comparable among HIV-positive and HIV-negative patients during transplant hospitalization. Infection and mortality rates were similar between both groups. | The study concludes that kidney transplantation is a viable option for HIV-positive patients, showing comparable outcomes with HIV-negative recipients in terms of clinical and fiscal aspects. | n/a |
| Azar2017(6) | USA | Retrospective cohort study | 13 | 0 | 1/1/2000 – 31/12/2015 | Single center | Integrase strand transferase inhibitor (ISTI)-based regimens vs. non-ISTI-based regimens | Cumulative survival was 75%  One-year and three-year survival rates were 100% and 63%  In ISTI-based regimens: 100% graft survival with two allograft rejections  In non-ISTI-based regimens: Graft failure in three patients | ISTI-based therapy, preferably instituted prior to transplantation, is the preferred antiretroviral regimen in HIV-positive renal transplantation. | n/a |
| Boyle2017(7) | USA | Retrospective Cohort Study | 104 | 0 | 2001 - 2014 | Single center | Tenofovir disoproxil fumarate (TDF) based antiretroviral therapy (ART) | Primary allograft failure at 3 years | TDF use following kidney transplantation was not significantly associated with primary allograft failure. | CoxPH |
| Boyle2020(8) | USA | Retrospective Observational Study | 42 | 0 | 1/1/2008 – 31/12/2015 | Single center | Characterization of barriers in kidney transplant evaluation in HIV-positive patients | Comorbidities, viral control and management, viral resistance, and kidney transplant evaluation appointment rates | HIV-positive patients face significant barriers in the kidney transplant evaluation process, especially those with psychiatric illnesses and viral resistance. | n/a |
| Camargo2019(9) | USA | Prospective observational cohort study | 22 | 0 | 8/2015 – 5/2017 | Single center | predictive value of pretransplant levels of inflammation (CRP and sTNF-R1) and immune activation markers (activated CD8+ T cells) for acute rejection in HIV-positive kidney transplant recipients. | Elevated pretransplant levels of sTNF-R1, CRP, and frequencies of activated CD8+ T cells were significantly higher in patients who experienced acute rejection. High pretransplant levels of these biomarkers were associated with an increased risk of acute rejection. | Pretransplant levels of certain biomarkers can help identify HIV-positive kidney transplant recipients at increased risk for acute rejection, potentially aiding in personalized immunosuppression regimens. | KM |
| Camargo2019a(10) | USA | Retrospective Cohort Study | 13 | 0 | 8/2007 – 8/2017 | Single center | Kidney transplant in HIV+/HCV+ coinfected recipients | 1-year patient survival, death-censored graft survival, acute rejection, serious infections | Patient and death-censored graft survivals at one year post-transplant were 92% and 85%, respectively. Serious infections were significantly higher in the pre-DAA group (67%) compared to the post-DAA group (0%). | KM |
| Delaney1992(11) | USA | Retrospective cohort study | 17 | 0 | 1976 - 1985 | Single center | retrospective analysis of mortality, morbidity, and graft survival in two groups of HIV-positive patients. Group 1 included nine patients seropositive for an indefinite period before transplantation, and Group 2 included six patients who seroconverted in the perioperative period. | Group 1 experienced graft loss due to chronic rejection, sepsis, and death with a functioning graft. Group 2 had two patients with functioning allografts at the time of the study, and causes of allograft loss and complications were similar to Group 1 but with less frequent acute rejection. | HIV infection in renal allograft recipients is associated with poor allograft survival, mostly due to chronic rejection and increased infections requiring hospitalization. | n/a |
| Durand2021(12) | USA | Prospective Multicenter Pilot Observational Study | 75 | 0 | 3/2016 – 7/2019 | Multi-center | HIV-positive donor to HIV-positive recipient (HIV D+/R+) kidney transplantation vs HIV-negative donor to HIV+ recipient (HIV D−/R+) | 1-year graft survival, estimated glomerular filtration rate (eGFR), HIV breakthrough, infectious hospitalizations, opportunistic infections, acute rejection | Overall transplant and HIV outcomes were excellent. Trend toward higher rejection with D+ raises concerns for further investigation. | CoxPH |
| Durand2024(13) | USA | Observational study | 198 | 0 | 4/2018 – 9/2021 | Multi-center | Comparison of kidney transplantation outcomes from HIV-positive and HIV-negative deceased donors in HIV-positive recipients | Transplantation from HIV-positive donors was noninferior to HIV-negative donors concerning safety events, with similar survival, graft loss, and rejection rates. | Demonstrated feasibility of expanding kidney transplantation from HIV-positive donors to clinical care. Slightly higher HIV breakthrough infections due to therapy nonadherence. | CoxPH |
| Frassetto2007(14) | USA | Observational study | 20 | 0 |  | Multi-center | investigated the pharmacokinetics and dosing modifications of immunosuppressants in HIV-1 infected liver and kidney transplant recipients who were on both immunosuppressants and antiretrovirals. | Significant drug interactions between antiretrovirals and immunosuppressants were noted, necessitating adjustments in immunosuppressant dosing. The study found marked increases in immunosuppressant trough levels in subjects on protease inhibitors, necessitating a reduction in dose or an increase in dosing interval. | Highlighted the complexity and importance of managing drug interactions and therapeutic drug monitoring in HIV-infected transplant recipients. It underscored the need for careful management to avoid insufficient immunosuppression or toxicity due to drug interactions. | n/a |
| Frassetto2013(15) | USA | Observational study | 26 | 0 |  | Single center | analyzed the pharmacokinetics of cyclosporine and tacrolimus in HIV-1 infected liver and kidney transplant recipients who were on both immunosuppressants and antiretrovirals (either protease inhibitors or non-nucleoside reverse transcriptase inhibitors). | Significant drug interactions between antiretrovirals and immunosuppressants, impacting drug bioavailability and clearance. These interactions necessitated adjustments in immunosuppressant dosing. | Highlighted the complexity and importance of therapeutic drug monitoring in HIV-infected transplant recipients due to significant drug interactions between antiretrovirals and immunosuppressants, affecting pharmacokinetics and necessitating dosage adjustments. | n/a |
| Gasser2009(16) | Multi-country (United States, Switzerland, Spain) | Prospective cohort study | 27 | 0 |  | Multi-center | impact of transplantation and immune-depleting treatment (including thymoglobulin) on CD4+ T-cell counts, viral loads, and virus-specific T-cell immunity, with a focus on the herpesviruses Epstein-Barr Virus (EBV) and Cytomegalovirus (CMV). | Significant depletion of polyfunctional virus-specific CTL (cytotoxic T lymphocyte) in individuals receiving thymoglobulin, accompanied by virologic EBV-reactivation events. The disappearance of polyfunctional CTL was directly linked to viral reactivation. | Immune-depleting treatments in HIV+ kidney transplant recipients can significantly impact immune functionality, particularly affecting the control over herpesvirus reactivation. Long-term studies are needed to assess the clinical risk associated with such treatments, especially concerning EBV-associated lymphoproliferative diseases. | n/a |
| Gathogo2014(17) | UK | National cohort study | 35 | 0 | 1/2005 – 12/2010 | Multi-center | investigated the outcomes of kidney transplantation in HIV-positive patients, focusing on patient survival, graft survival, acute rejection, and clinical complications including HIV disease progression. | Patient survival rates of 91.3% at 1 and 3 years, and graft survival rates of 91.3% and 84.7% respectively. The cumulative incidence of acute rejection was high at 48%, and there were instances of transient HIV viremia and declines in CD4 cell counts post-transplantation. | Feasibility of kidney transplantation in HIV-positive patients with well-controlled viral replication on antiretroviral therapy. It reported favorable patient and graft survival rates but noted a high incidence of acute rejection, indicating that the optimal immune suppression strategy in this population remains to be refined. | KM |
| Gathogo2016(18) | UK | National observational cohort study | 78 | 0 | 1/1/2005 – 31/12/2013 | Multi-center | efficacy and safety of Cyclosporin (CsA) and Tacrolimus (Tac) based immunosuppressive therapy in HIV positive kidney transplant recipients. | Acute allograft rejection (AR) was significantly more common among patients who started on CsA (58%) compared with those on Tac (21%). The cumulative incidence of AR at 1 year was 60% in the CsA group compared with 20% in the Tac group. | The study concluded that Tacrolimus may be the preferred calcineurin inhibitor for use in kidney transplantation in people living with HIV, due to its association with a lower risk of acute allograft rejection compared to Cyclosporin. | CoxPH |
| Gathogo2017(19) | UK | Analysis of registry data | 51 | 0 | 1/2005 – 12/2013 | UK transplant registry | kidney transplant outcomes in HIV serodiscordant recipient pairs. It analyzed data from the UK transplant registry and compared donor and recipient characteristics, as well as patient and graft survival rates. | Similar rates of delayed graft function and overall survival rates for HIV-positive and HIV-negative recipients. However, HIV-positive recipients experienced a higher incidence of acute graft rejection within the first year post-transplantation. | Long-term transplant outcomes in HIV-positive recipients are not as favorable as in non-infected individuals. It also indicates that HIV infection, black ethnicity, and greater HLA mismatch are associated with increased allograft rejection. These findings underscore the need for carefully tailored immunosuppression strategies in HIV-positive kidney transplant recipients. | CoxPH |
| Izzo2018(20) | Itaaly | Retrospective Observational Single-Center Study | 28 | 0 | 1/2005 – 10/2016 | Single center | Kidney transplantation in HIV-infected patients | CD4+ count, HIV RNA undetectability, acute rejections, patient survival | Kidney transplantation is safe in carefully selected HIV-infected patients. High incidence of acute rejection observed; optimization of immunosuppressive therapy needed. | n/a |
| Kucirka2016(21) | USA | Prospective Observational Study | 830 | 0 | 1/1/2000 – 1/12/2014 | Scientific RegIstry of Transplant Recipients | Comparison of outcomes with different induction immunosuppression treatments (anti-thymocyte globulin [ATG], IL-2 receptor blocker, and no induction) | Evaluation of delayed graft function (DGF), acute rejection (AR), graft loss, and death  Infections and hospitalizations also assessed in a subset of 308 patients with Medicare | Induction immunosuppression, particularly with ATG, was associated with lower rates of AR and not with increased infection risk  Patients receiving induction had fewer hospitalization days, lower rates of DGF, less graft loss, and a trend toward lower mortality | CoxPH |
| Locke2009(22) | USA | Retrospective analysis | 100 | 36492 | 1/1/2004 – 30/6/2006 | United Network for Organ Sharing | long-term outcomes and risk factors for graft loss in renal transplant among HIV-positive patients. | Lower long-term allograft survival among HIV-positive recipients, with controllable risk factors possibly explaining this disparity. Proper donor selection and transplant recipient management, including avoiding prolonged cold ischemic time, using living donors, and determining optimal immunosuppression dosing before transplant, could achieve long-term graft survival comparable to that in HIV-negative patients. | With appropriate management and donor selection, HIV-positive patients can achieve comparable long-term graft survival to HIV-negative patients, though they face higher risks and challenges. | KM |
| Locke2014(23) | USA | Retrospective Cohort Study | 516 | 93027 | 1/11/2003 31/12/2011 | Scientific RegIstry of Transplant Recipients | Kidney transplantation with different immunosuppression regimens | Acute rejection (AR) within 1 year, patient survival, death-censored graft survival (DCGS) | ATG induction associated with a lower risk of AR in HIV-positive KT recipients. Sirolimus-based maintenance therapy linked to a higher risk of AR. | CoxPH |
| Locke2015(24) | USA | Observational study | 499 | 0 | 1/1/2004 – 31/12/2011 | Scientific RegIstry of Transplant Recipients | influence of center-level experience and participation in the NIH consortium on transplant outcomes in HIV-infected recipients, using SRTR data from 2004-2011. It analyzed associations between center experience measures and outcomes. | No difference in outcomes among centers early in their experience compared to more experienced centers. Participation in the NIH study was not associated with better outcomes. Transplants performed in the more recent era (2008-2011) showed significantly better outcomes than those in the earlier era (2004-2007). | Transplant era was strongly associated with outcomes, suggesting that improvements in HIV care, particularly the introduction of new antiretroviral therapies, played a significant role in enhancing transplant outcomes over time. It supports the continued expansion of HIV-positive kidney transplantation in the United States. | CoxPH |
| Locke2016(25) | USA | Retrospective Cohort Study | 526 | 82236 | 1/1/2001 – 31/12/2013 | Scientific RegIstry of Transplant Recipients | Analysis of donor–recipient combinations | Graft loss in HIV-positive kidney transplant recipients | HCV infection and more than three HLA mismatches significantly amplify the risk of graft loss associated with HIV infection. Monoinfected HIV-positive recipients with fewer HLA mismatches had a graft loss risk comparable to uninfected counterparts. | CoxPH |
| Lorio2016(26) | USA | Retrospective Cohort Study | 36 | 0 | 10/2006 – 9/2013 | Single center | Assessment of pre-transplant immune activation levels on acute rejection | Allograft rejection rates in relation to T cell activation levels | High levels of pre-transplant T cell activation did not correspond with an increased risk of allograft rejection. Paradoxically, those with the highest levels of immune activation had a lower risk of rejection. | KM |
| Malat2018(27) | USA | Retrospective Observational Study | 120 | 0 |  | Single center | Kidney transplantation in HIV-positive patients | Patient and graft survival, rejection rates, management of ART, donor selection, post-transplantation surveillance | Kidney transplantation in HIV-positive patients has evolved from high-risk to standard care with comparable outcomes to HIV-negative recipients. Challenges remain in access to transplantation and high ABMR rates. | KM |
| Martina2011(28) | Spain | Prospective Study | 11 | 0 | 2005 – 2011 | Single center | Renal transplantation in HIV-infected patients with ESRD | Patient survival, graft survival, acute tubular necrosis, and acute rejection episodes | Renal transplantation in adequately selected HIV-infected patients is safe with acceptable patient and graft survivals, similar to HIV-negative recipients. | n/a |
| Matignon2019(29) | France | Prospective multicentre single-arm trial | 26 | 174 | 12/2011 – 12/2013 | Multi-center | ANRS 153 TREVE, was focused on assessing the incidence of acute rejection within 6 months of kidney transplantation in HIV-infected recipients treated with a protease-inhibitor-free raltegravir-based regimen. | Low incidence of acute rejection (8% at 6 and 12 months) in the participants. It also noted a median time to transplantation longer in people living with HIV (PLHIV) than in controls, underscoring poorer access to transplantation. | Kidney transplantation in PLHIV treated with a raltegravir-based regimen is associated with low rates of acute rejection. However, PLHIV have poorer access to transplantation compared to uninfected individuals. | CoxPH |
| Mazuecos2011(30) | Spain | Multicenter retrospective case–control study | 20 | 40 | 2001 – 2009 | Multi-center | Renal transplant in HIV+ patients compared with HIV- controls | Graft survival, incidence of acute rejection | HIV+ recipients had a lower incidence of immediate renal function and a higher rate of acute rejection. Graft survival was slightly lower in HIV+ patients, with coinfection with HCV significantly impacting graft survival. Overall, outcomes were good, particularly in non-coinfected patients. | KM |
| Mazuecos2012(31) | Spain | Cross-sectional and retrospective cohort study | 10 | 0 | 2001 – 2011 | SICATA Registry | Analysis of renal replacement therapy (RRT) modalities and renal transplantation outcomes | Renal transplantation safety and outcomes in HIV-infected patients | Renal transplantation was found to be safe in correctly selected HIV-infected patients. The study highlighted the low number of HIV-infected patients on the transplantation waiting list, possibly due to high comorbidity or lack of systematic assessment for transplantation. | KM |
| Mazuecos2013(32) | Spain | Multicenter retrospective cohort study | 36 | 72 | 1/2001 – 12/2011 | Multi-center | analyzed kidney transplant outcomes in HIV-infected patients in Spain from 2001 to 2011, focusing on early post-transplant complications such as acute rejection and delayed graft function (DGF). | Higher incidence of DGF in HIV-infected recipients compared to HIV-negative recipients. Graft survival at one and three years was slightly lower in HIV-infected patients, though HIV infection was not a significant predictor of graft loss when adjusted for other factors. | While HIV-infected kidney transplant recipients experience a higher incidence of early complications like DGF, their overall graft survival is comparable to HIV-negative recipients. The results underline the importance of careful management and monitoring in this patient group. | CoxPH |
| MorenoRamirez2020(33) | Spain | Retrospective Cohort Study | 11 | 0 | 2001 – 2018 | Single center | Renal transplantation in HIV-HCV coinfected patients with direct-acting antivirals treatment | Sustained virologic response, improvement of liver function, renal function, HIV viral load control | Direct-acting antivirals therapy is safe and effective for patients with HIV-HCV coinfection, offering new treatment possibilities. | n/a |
| Muller2015(34) | South Africa | Prospective Nonrandomized Study | 27 | 0 | 9/2008 – 2/2014 | Single center | Kidney transplantation from HIV-positive deceased donors to HIV-positive recipients | Patient survival, graft survival, acute rejection | Kidney transplantation from HIV-positive donors is a viable treatment option for HIV-infected patients needing renal-replacement therapy. | KM |
| Muller2018(35) | South Africa | Observational study | 43 | 0 | 2008 – 2016 | Single center | Analysis of outcomes of transplanting HIV-positive kidneys into HIV-positive recipients. | Successful transplant outcomes with considerations around HIV superinfection and resistance. It highlighted the effective use of HIV-positive donors in a setting with limited resources and high HIV prevalence. | Transplanting HIV-positive kidneys into HIV-positive recipients can be a viable option, especially in resource-limited settings with a high prevalence of HIV, albeit with certain risks and considerations. | n/a |
| Muthukumar2013(36) | USA | Retrospective cohort study | 11 | 0 | 5/2006 – 5/2010 | Single center | outcomes of HIV-infected kidney graft recipients managed with an early corticosteroid withdrawal protocol | The incidence of acute rejection was 9% at 1 year. Patient and allograft survival rates were 100% and 91% respectively. No progression of HIV or BKV replication was observed. | An early steroid withdrawal regimen with antithymocyte globulin induction is associated with excellent graft and patient outcomes in HIV-infected recipients of kidney allografts. Their urinary cell mRNA profiles were similar to those of HIV-negative patients with stable graft function. | KM |
| Qiu2006(37) | USA | Retrospective analysis using United Network for Organ Sharing kidney transplant data | 38 | 38 | 1997 – 2004 | United Network for Organ Sharing | Kidney transplant in HIV+ patients compared to HIV- patients from the same donors | Graft and patient survival | HIV+ renal recipients achieved graft and patient survival rates similar to HIV- recipients, supporting the suitability of transplantation in HIV+ patients. | KM |
| Roland2008(38) | USA | Prospective nonrandomized trial | 18 | 0 | 3/2000 – 9/2003 | Multi-center | Liver or kidney transplant in patients with stable HIV disease | 1- and 3-year survival rates for liver recipients: 91% and 64%, respectively 1- and 3-year survival rates for kidney recipients: 94% (specific data for 3 years not mentioned) Liver graft survival: 82% at 1 year and 64% at 3 years Kidney graft survival: 83% (specific time frame not mentioned) | The document doesn’t explicitly state the conclusions on the first page, further analysis of the document is needed to extract this information. | CoxPH |
| Roland2016(39) | USA | Observational Cohort Study | 150 | 85153 | 10/2003 – 6/2009 | Scientific RegIstry of Transplant Recipients | Evaluation of liver and kidney transplantation in HIV-positive recipients compared to HIV-negative controls | Survival benefit, death and graft loss, opportunistic and non-opportunistic infections | Kidney transplantation recommended for well-managed HIV-positive patients. Liver transplantation beneficial for candidates with high MELD. Similar outcomes between HIV-positive and HIV-negative recipients. | CoxPH |
| Sawinski2015(40) | USA | Retrospective Observational Study | 492 | 117791 | 1/10/1987 – 31/12/2013 | United Network for Organ Sharing | Comparison of patient and allograft outcomes among these groups | Patient and allograft survival rates in HIV patients did not significantly differ from the uninfected reference group | The study suggests comparable or superior outcomes in HIV-infected kidney transplant patients compared to HCV-infected or HIV/HCV-coinfected recipients | CoxPH |
| Sawinski2017(41) | USA | Retrospective cohort study | 332 | 0 | 1/1/2001 – 1/10/2012 | United Network for Organ Sharing | impact of protease inhibitor–based anti-retroviral therapy (ART) compared to non–protease inhibitor-based ART on the outcomes of HIV-positive kidney transplant recipients. | Protease inhibitor-based ART was associated with a 1.8-fold increased risk of allograft loss and a 1.9-fold increased risk of death compared to non-protease inhibitor regimens. | Recipients should be converted to a non-protease inhibitor regimen prior to kidney transplantation due to the increased risks associated with protease inhibitor-based regimens. | CoxPH |
| Shelton2017(42) | USA | Retrospective Cohort Study | 22 | 4127 | 2004 - 2013 | United Network for Organ Sharing | Kidney retransplantation in HIV+ individuals | Mortality and graft loss in HIV+ re-KT recipients compared to HIV- recipients | HIV+ re-KT recipients had a 3.11-fold increased risk of death and a 1.96-fold increased risk of graft loss compared to HIV- re-KT recipients. Coinfection with HCV significantly amplified the risk of graft loss. | CoxPH |
| Stock2003(43) | USA | Pilot safety and efficacy trial | 10 | 0 |  | Single center | safety and efficacy of kidney and liver transplantation in patients infected with HIV. It included patients with stable HIV disease, using a combination of antiretroviral therapies. | All kidney transplant recipients and three out of four liver transplant recipients were alive with functioning grafts at the time of follow-up. Rejection occurred in 5 of the 10 kidney transplant recipients. HIV viral loads remained undetectable in all patients maintained on HAART. | Transplantation in HIV-infected patients with well-controlled disease is safe and effective. It indicated that HIV should no longer be considered an absolute contraindication to solid-organ transplantation. | n/a |
| Stock2010(44) | USA | Prospective nonrandomized trial | 150 | 31110 | 11/2003 – 6//2009 | Multi-center | kidney transplantation in HIV-infected candidates with CD4+ T-cell counts of at least 200 per cubic millimeter and undetectable plasma HIV type 1 RNA levels while being treated with a stable antiretroviral regimen. It included interventions like prophylaxis against opportunistic infection, indications for biopsy, and immunosuppression management. | Patient survival rates at 1 and 3 years were 94.6% and 88.2%, respectively, and graft survival rates were 90.4% and 73.7%. A high rejection rate was observed, with 1-year and 3-year estimates of 31% and 41%, respectively. HIV infection remained well controlled with stable CD4+ T-cell counts. | Feasibility of kidney transplantation in carefully selected HIV-infected patients, demonstrating high patient and graft survival rates. However, it also indicated a need for better immunotherapy due to unexpectedly high rejection rates. | KM |
| Stock2014(45) | USA | Prospective Cohort Study | 91 | 0 |  | Multi-center | Analysis of the impact of immunosuppressant therapy on HIV persistence | Levels of HIV RNA and DNA in plasma and cells post-transplant | Sirolimus use post-transplantation was associated with lower HIV DNA levels, suggesting potential beneficial effects on HIV persistence and supporting future studies of sirolimus as a reservoir-modifying agent. | n/a |
| Touzot2010(46) | France | Retrospective cohort study | 27 | 0 | 3/2005 – 1/2009 | Multi-center | induction therapy and long-term treatment combining mycophenolate mofetil, steroids, and either tacrolimus or cyclosporine | 100% patient survival rate at 1 year and a 98% rate at 2 years. Graft survival was 98% at 1 year and 96% at 2 years. The mean glomerular filtration rate values at 12 and 24 months were 60.6 and 65.4 mL/min/1.73 m², respectively. Acute cellular rejection was diagnosed in 15% of the cases. | The study confirmed the safety of renal transplantation in HIV-infected patients with few adverse events and a low incidence of acute rejection | n/a |
| Vicari2016(47) | Brazil | Case–control study | 53 | 106 | 2/2006 – 7/2014 | Multi-center | compared the outcomes of HIV-infected kidney transplant recipients with HIV-negative controls. Delayed graft function, acute rejection, infections, graft function, and survival. | HIV-positive recipients exhibited a higher incidence of treated acute rejection and delayed graft function. One-year patient survival and graft survival were lower but acceptable in the HIV-infected group. | Despite somewhat inferior outcomes, kidney transplantation is an adequate therapy for selected HIV-infected recipients in Brazil. | CoxPH |
| Vivanco2013(48) | USA | Retrospective analysis of registry data | 88 | 4822 | 1/1/2003 – 31/12/2010 | Scientific RegIstry of Transplant Recipients | outcomes of different induction therapies in hepatitis C virus (HCV) positive kidney transplant recipients, focusing on Alemtuzumab (AZ) compared to other therapies. | Induction therapy was associated with significantly better overall patient survival and death-censored graft survival, compared to no induction. Specifically, AZ, other T-cell depleting agents, and IL-2Rab showed benefits over no induction. | Induction therapy, including Alemtuzumab, is associated with better patient and death-censored graft survival in HCV-positive kidney transplant recipients compared to noninduction. In HCV/HIV coinfected patients, induction therapy is not contraindicated and may be beneficial. | CoxPH |
| Yoon2011(49) | USA | Retrospective cohort study | 251 | 149867 | 1/1/1995 – 29/9/2006 | United States Renal Data System | trends in renal transplantation among patients with human immunodeficiency virus (HIV) infection, analyzing data from the United States Renal Data System. | Significant increase in renal transplant recipients who were HIV seropositive, with more African American recipients and a higher prevalence of hepatitis C virus coinfection. Improved access to transplant wait listing and better management of immunosuppression were observed, particularly among African American patients. | Dramatic increase in the number of transplants among HIV-infected patients, suggesting improved access to transplantation and better management of immunosuppression, especially among African American patients. | CoxPH |

CoxPH: Cox Proportional Hazard; KM: Kaplan Meier survival analysis

##### Supplementary Table S2: Data extracted for synthesis and analysis of primary outcomes

|  |  |  | 1-year patient survival (%) | | 3-year patient survival (%) | | >= 5-year patient survival (%) | | 1-year graft survival (%) | | 3-year graft survival (%) | | >= 5-year graft survival (%) | | 1-year cumulative graft rejection (%) | | 3-year cumulative graft rejection (%) | | >= 5-year cumulative graft rejection (%) | | 1-year infection incidence(100 pt-yr) | | 3-year infection incidence(100 pt-yr) | | >= 5-year infection incidence(100 pt-yr) | |
| --- | --- | --- | --- | --- | --- | --- | --- | --- | --- | --- | --- | --- | --- | --- | --- | --- | --- | --- | --- | --- | --- | --- | --- | --- | --- | --- |
| Study ID | **HIV +** | **HIV -** | **HIV+** | **HIV-** | **HIV+** | **HIV-** | **HIV+** | **HIV-** | **HIV+** | **HIV-** | **HIV+** | **HIV-** | **HIV+** | **HIV-** | **HIV+** | **HIV-** | **HIV+** | **HIV-** | **HIV+** | **HIV-** | **HIV+** | **HIV-** | **HIV+** | **HIV-** | **HIV+** | **HIV-** |
| Abbott2004 | 47 | 27804 |  |  | 95.74 | 87.17 |  |  |  |  | 97.7 | 93.2 |  |  |  |  |  |  |  |  |  |  |  |  |  |  |
| Ailioaie2017 | 24 | 21 |  |  |  |  | 91.67 | 100 |  |  |  |  | 87.5 | 80.95 |  |  |  |  |  |  |  |  |  |  | 24 | 27 |
| Alfano2018 | 19 | 200 | 94.4 | 98.9 | 94.4 | 95.6 | 70.8 | 92.9 | 89.5 | 91.4 | 77.1 | 82.1 | 57.8 | 78.3 | 32.90 |  | 40.3 |  | 48.8 |  |  |  |  |  | 26.32 |  |
| Alfano2020 | 22 | 0 |  |  |  |  | 63.64 |  |  |  |  |  | 81.82 |  |  |  |  |  |  |  |  |  |  |  |  |  |
| Apewokin2018 | 605 | 103532 |  |  |  |  |  |  |  |  |  |  |  |  |  |  |  |  | 27.5 | 20.75 |  |  |  |  |  |  |
| Azar2017 | 13 | 0 | 100 |  | 63 |  |  |  | 100 |  | 100 |  |  |  | 15.38 |  | 23.08 |  |  |  |  |  |  |  |  |  |
| Boyle2017 | 104 | 0 |  |  | 85.58 |  |  |  |  |  | 57.69 |  |  |  | 66.35 |  |  |  |  |  |  |  |  |  |  |  |
| Boyle2020 | 42 | 0 |  |  |  |  |  |  |  |  |  |  |  |  |  |  |  |  |  |  |  |  |  |  |  |  |
| Camargo2019 | 22 | 0 | 100 |  |  |  |  |  | 91 |  |  |  |  |  |  |  | 13.64 |  |  |  |  |  | 7.58 |  |  |  |
| Camargo2019a | 13 | 0 | 92 |  |  |  |  |  | 85 |  |  |  |  |  | 7.69 |  |  |  |  |  | 30.77 |  |  |  |  |  |
| Delaney1992 | 17 | 0 |  |  | 88.24 |  | 47.06 |  | 94.12 |  | 51.76 |  | 25.88 |  | 5.88 |  | 41.18 |  | 58.82 |  |  |  |  |  |  |  |
| Durand2021 | 75 | 0 | 100 |  |  |  |  |  | 91.71 |  |  |  |  |  | 36.00 |  |  |  |  |  |  |  |  |  | 15 |  |
| Frassetto2007 | 20 | 0 |  |  |  |  |  |  |  |  |  |  |  |  |  |  |  |  |  |  |  |  |  |  |  |  |
| Durand2024 | 198 | 0 |  |  | 94.5 |  | 86 |  |  |  | 97.47 |  | 94.95 |  |  |  | 17 |  | 22.5 |  |  |  |  |  |  |  |
| Frassetto2013 | 26 | 0 |  |  |  |  |  |  |  |  |  |  |  |  |  |  |  |  |  |  |  |  |  |  |  |  |
| Gasser2009 | 27 | 0 |  |  |  |  |  |  | 100 |  |  |  |  |  | 33.33 |  |  |  |  |  |  |  |  |  |  |  |
| Gathogo2014 | 35 | 0 | 91.3 |  | 91.3 |  |  |  | 91.3 |  | 84.7 |  |  |  | 47.00 |  |  |  |  |  |  |  |  |  | 12 |  |
| Gathogo2016 | 78 | 0 | 96.8 |  |  |  |  |  | 95.3 |  |  |  |  |  | 35.90 |  |  |  |  |  |  |  |  |  |  |  |
| Gathogo2017 | 51 | 0 | 98 | 100 | 91 | 90 | 91 | 90 | 93 | 100 | 78 | 100 | 78 | 100 | 36.00 | 24 |  |  |  |  |  |  |  |  |  |  |
| Izzo2018 | 28 | 0 |  |  |  |  | 82.1 |  |  |  |  |  | 71.4 |  |  |  |  |  | 57.1 |  | 57.1 |  |  |  |  |  |
| Kucirka2016 | 830 | 0 |  |  |  |  |  |  |  |  |  |  |  |  |  |  |  |  |  |  |  |  |  |  |  |  |
| Locke2009 | 100 | 36492 | 95.4 | 96.2 |  |  |  |  | 87.9 | 94.6 |  |  |  |  |  |  |  |  |  |  |  |  |  |  |  |  |
| Locke2014 | 516 | 93027 | 98 | 96.6 |  |  |  |  | 91.7 | 94.2 |  |  |  |  | 15.00 | 8 |  |  |  |  |  |  |  |  |  |  |
| Locke2015 | 499 | 0 | 94.56 |  | 87.64 |  | 82.89 |  | 88.55 |  | 77.7 |  | 69.35 |  |  |  |  |  |  |  |  |  |  |  |  |  |
| Locke2016 | 526 | 82236 |  |  |  |  |  |  |  |  |  |  |  |  |  |  |  |  |  |  |  |  |  |  |  |  |
| Lorio2016 | 36 | 0 |  |  |  |  |  |  |  |  |  |  |  |  | 17.87 |  | 22.64 | 14 |  |  |  |  |  |  |  |  |
| Malat2018 | 120 | 0 | 96.67 |  | 93.33 |  |  |  | 88.33 |  | 61.67 |  |  |  | 53.33 |  |  |  | 66.67 |  |  |  |  |  |  |  |
| Martina2011 | 11 | 0 | 100 |  | 100 |  | 100 |  |  |  |  |  |  |  |  |  |  |  |  |  |  |  |  |  |  |  |
| Matignon2019 | 26 | 174 | 92.31 |  | 89 | 96 |  |  |  |  | 82 | 83 |  |  | 8.00 |  |  |  |  |  |  |  |  |  |  |  |
| Mazuecos2011 | 20 | 40 | 100 | 100 | 100 | 100 | 95 | 100 | 85 | 97.5 | 74.4 | 97.5 | 74.4 | 91 |  |  |  |  | 40 | 22.5 |  |  |  |  | 8.75 | 4.69 |
| Mazuecos2012 | 10 | 0 | 100 |  | 100 |  | 100 |  | 100 |  | 100 |  | 75 |  |  |  |  |  | 20 |  |  |  |  |  |  |  |
| Mazuecos2013 | 36 | 72 | 100 | 100 | 100 | 100 | 91.67 | 97.22 | 91.6 | 97.1 | 86.2 | 94.7 |  |  |  |  |  |  | 27.78 | 5.56 |  |  |  |  | 6.94 | 0.97 |
| MorenoRamirez2020 | 11 | 0 |  |  |  |  |  |  |  |  |  |  |  |  |  |  |  |  |  |  |  |  |  |  |  |  |
| Muller2015 | 27 | 0 | 84 | 91 | 84 |  | 74 | 85 | 93 | 88 | 84 |  | 84 | 75 | 8.00 |  | 22 |  |  |  |  |  |  |  |  |  |
| Muller2018 | 43 | 0 |  |  |  |  |  |  |  |  |  |  |  |  |  |  |  |  |  |  |  |  |  |  |  |  |
| Muthukumar2013 | 11 | 0 | 100 |  | 90 |  |  |  | 91 |  | 81 |  |  |  | 9.00 |  | 18 |  |  |  | 54.55 |  |  |  |  |  |
| Qiu2006 | 38 | 38 |  |  |  |  | 87.3 | 91.3 |  |  |  |  | 65.1 | 76.1 | 10.53 | 10.53 |  |  |  |  |  |  |  |  |  |  |
| Roland2008 | 18 | 0 | 94 | 95.9 | 94 | 90.8 |  |  | 83 | 91.9 | 83 | 82.4 |  |  | 52.00 |  | 73 |  |  |  |  |  |  |  |  |  |
| Roland2016 | 150 | 85153 |  |  |  |  | 88.7 | 87.2 |  |  |  |  | 69.3 | 75.6 |  |  |  |  |  |  | 131.33 |  |  |  |  |  |
| Sawinski2015 | 492 | 117791 |  |  | 89 | 90 |  |  |  |  | 81 | 86 |  |  |  |  |  |  | 19.24 |  |  |  |  |  |  |  |
| Sawinski2017 | 332 | 0 |  |  | 94.58 |  |  |  |  |  | 87.1 |  |  |  |  |  |  |  | 18.5 |  |  |  |  |  |  |  |
| Shelton2017 | 22 | 4127 |  |  | 80.2 | 92.1 |  |  |  |  | 66.7 | 82.7 |  |  |  |  |  |  |  |  |  |  |  |  |  |  |
| Stock2003 | 10 | 0 | 100 |  |  |  |  |  |  |  |  |  |  |  |  |  |  |  | 50 |  | 50 |  |  |  |  |  |
| Stock2010 | 150 | 31110 | 94.6 | 96.2 | 88.2 | 90.6 |  |  | 90.4 | 92.5 | 73.7 | 82.8 |  |  | 31.00 | 12.3 | 41 |  |  |  |  |  |  |  | 16.73 |  |
| Stock2014 | 91 | 0 |  |  |  |  |  |  |  |  |  |  |  |  |  |  |  |  |  |  |  |  |  |  |  |  |
| Touzot2010 | 27 | 0 | 100 |  |  |  |  |  | 98 |  |  |  |  |  | 15.00 |  |  |  |  |  |  |  |  |  | 30.94 |  |
| Vicari2016 | 53 | 106 | 90.6 | 100 |  |  |  |  | 90.4 | 98.1 |  |  |  |  |  |  |  |  | 49.1 | 31.1 |  |  |  |  | 22.57 | 13.3 |
| Vivanco2013 | 88 | 4822 | 88.64 | 92.18 |  |  |  |  |  |  |  |  |  |  | 18.20 | 8.5 |  |  |  |  |  |  |  |  |  |  |
| Yoon2011 | 251 | 149867 |  |  |  |  |  |  |  |  |  |  |  |  |  |  |  |  |  |  |  |  |  |  |  |  |

##### Supplementary Table S3: Heterogeneity analysis

|  | Patient Survival | | | Graft Survival | | | Graft Rejection | | | Infection |  |  | Patient Survival | |  | Graft Survival | |  | Graft Rejection | |  |  |
| --- | --- | --- | --- | --- | --- | --- | --- | --- | --- | --- | --- | --- | --- | --- | --- | --- | --- | --- | --- | --- | --- | --- |
| Study ID | 1 year | 3 years | >= 5 years | 1 year | 3 years | >= 5 years | 1 year | 3 years | >= 5 years | 1 year | 3 years | >= 5 years | 1 year | 3 years | >= 5 years | 1 year | 3 years | >= 5 years | 1 year | 3 years | >= 5 years | Total |
| DATA_STARTS_HERE |  |  |  |  |  |  |  |  |  |  |  |  |  |  |  |  |  |  |  |  |  | 0 |
| Abbott2004 |  |  |  |  |  |  |  |  |  |  |  |  |  | * |  |  | * |  |  |  |  | 2 |
| Ailioaie2017 |  |  |  |  |  |  |  |  |  |  |  |  |  |  |  |  |  | * |  |  |  | 1 |
| Alfano2018 |  |  | * |  |  | * |  |  |  |  |  |  | * |  |  |  |  |  |  |  |  | 3 |
| Alfano2020 |  |  | * |  |  |  |  |  |  |  |  |  |  |  |  |  |  |  |  |  |  | 1 |
| Apewokin2018 |  |  |  |  |  |  |  |  |  |  |  |  |  |  |  |  |  |  |  |  | * | 1 |
| Azar2017 |  |  |  |  |  |  |  |  |  |  |  |  |  |  |  |  |  |  |  |  |  | 0 |
| Boyle2017 |  |  |  |  | * |  | * |  |  |  |  |  |  |  |  |  |  |  |  |  |  | 2 |
| Boyle2020 |  |  |  |  |  |  |  |  |  |  |  |  |  |  |  |  |  |  |  |  |  | 0 |
| Camargo2019 |  |  |  |  |  |  |  |  |  |  |  |  |  |  |  |  |  |  |  |  |  | 0 |
| Camargo2019a |  |  |  |  |  |  |  |  |  |  |  |  |  |  |  |  |  |  |  |  |  | 0 |
| Delaney1992 |  |  |  |  |  | * |  |  | * |  |  |  |  |  |  |  |  |  |  |  |  | 2 |
| Durand2021 |  |  |  |  |  |  |  |  |  |  |  |  |  |  |  |  |  |  |  |  |  | 0 |
| Durand2024 |  |  |  |  |  |  |  |  |  |  |  |  |  |  |  |  |  |  |  |  |  | 0 |
| Frassetto2007 |  |  |  |  |  |  |  |  |  |  |  |  |  |  |  |  |  |  |  |  |  | 0 |
| Frassetto2013 |  |  |  |  |  |  |  |  |  |  |  |  |  |  |  |  |  |  |  |  |  | 0 |
| Gasser2009 |  |  |  |  |  |  |  |  |  |  |  |  |  |  |  |  |  |  |  |  |  | 0 |
| Gathogo2014 |  |  |  |  |  |  |  |  |  |  |  |  |  |  |  |  |  |  |  |  |  | 0 |
| Gathogo2016 |  |  |  |  |  |  |  |  |  |  |  |  |  |  |  |  |  |  |  |  |  | 0 |
| Gathogo2017 |  |  |  |  |  |  |  |  |  |  |  |  |  |  |  |  | * | * |  |  |  | 2 |
| Izzo2018 |  |  |  |  |  |  |  |  |  |  |  |  |  |  |  |  |  |  |  |  |  | 0 |
| Kucirka2016 |  |  |  |  |  |  |  |  |  |  |  |  |  |  |  |  |  |  |  |  |  | 0 |
| Locke2009 |  |  |  |  |  |  |  |  |  |  |  |  |  |  |  |  |  |  |  |  |  | 0 |
| Locke2014 |  |  |  |  |  |  | * |  |  |  |  |  | * |  |  | * |  |  |  |  |  | 3 |
| Locke2015 |  |  |  |  |  |  |  |  |  |  |  |  |  |  |  |  |  |  |  |  |  | 0 |
| Locke2016 |  |  |  |  |  |  |  |  |  |  |  |  |  |  |  |  |  |  |  |  |  | 0 |
| Lorio2016 |  |  |  |  |  |  |  |  |  |  |  |  |  |  |  |  |  |  |  |  |  | 0 |
| Malat2018 |  |  |  |  |  |  | * |  |  |  |  |  |  |  |  |  |  |  |  |  |  | 1 |
| Martina2011 |  |  |  |  |  |  |  |  |  |  |  |  |  |  |  |  |  |  |  |  |  | 0 |
| Matignon2019 |  |  |  |  |  |  |  |  |  |  |  |  |  | * |  |  |  |  |  |  |  | 1 |
| Mazuecos2011 |  |  |  |  |  |  |  |  |  |  |  |  |  |  |  |  | * |  |  |  |  | 1 |
| Mazuecos2012 |  |  |  |  |  |  |  |  |  |  |  |  |  |  |  |  |  |  |  |  |  | 0 |
| Mazuecos2013 |  |  |  |  |  |  |  |  |  |  |  |  |  |  |  |  |  |  |  |  | * | 1 |
| MorenoRamirez2020 |  |  |  |  |  |  |  |  |  |  |  |  |  |  |  |  |  |  |  |  |  | 0 |
| Muller2015 |  |  |  |  |  |  |  |  |  |  |  |  |  |  |  | * |  | * |  |  |  | 2 |
| Muller2018 |  |  |  |  |  |  |  |  |  |  |  |  |  |  |  |  |  |  |  |  |  | 0 |
| Muthukumar2013 |  |  |  |  |  |  |  |  |  |  |  |  |  |  |  |  |  |  |  |  |  | 0 |
| Qiu2006 |  |  |  |  |  |  |  |  |  |  |  |  |  |  |  |  |  |  |  |  |  | 0 |
| Roland2008 |  |  |  |  |  |  |  | * |  |  |  |  |  |  |  |  |  |  |  |  |  | 1 |
| Roland2016 |  |  |  |  |  |  |  |  |  | * |  |  |  |  | * |  |  |  |  |  |  | 2 |
| Sawinski2015 |  |  |  |  |  |  |  |  | * |  |  |  |  |  |  |  |  |  |  |  |  | 1 |
| Sawinski2017 |  |  |  |  |  |  |  |  |  |  |  |  |  |  |  |  |  |  |  |  |  | 0 |
| Shelton2017 |  |  |  |  |  |  |  |  |  |  |  |  |  |  |  |  |  |  |  |  |  | 0 |
| Stock2003 |  |  |  |  |  |  |  |  |  |  |  |  |  |  |  |  |  |  |  |  |  | 0 |
| Stock2010 |  |  |  |  |  |  |  | * |  |  |  |  |  |  |  |  |  |  | * |  |  | 2 |
| Stock2014 |  |  |  |  |  |  |  |  |  |  |  |  |  |  |  |  |  |  |  |  |  | 0 |
| Touzot2010 |  |  |  |  |  |  |  |  |  |  |  |  |  |  |  |  |  |  |  |  |  | 0 |
| Vicari2016 |  |  |  |  |  |  |  |  | * |  |  |  | * |  |  | * |  |  |  |  |  | 3 |
| Vivanco2013 |  |  |  |  |  |  |  |  |  |  |  |  |  |  |  |  |  |  |  |  |  | 0 |
| Yoon2011 |  |  |  |  |  |  |  |  |  |  |  |  |  |  |  |  |  |  |  |  |  | 0 |

##### Supplementary Table S4: Sensitivity analysis of primary and secondary outcomes according to year of kidney transplantation

| Outcome Measure | Time Frame | 1997 - 2006 | | | Post 2007 | | |
| --- | --- | --- | --- | --- | --- | --- | --- |
|  |  | **Survival / Rate (95%CI)(%)** | **I^2^ (%)** | **P-Value** | **Survival / Rate (%)** | **I^2^ (%)** | **P-Value** |
| Patient Survival | 1 Year | 94.8 (91.78 – 96.70) | 0.00 | 1.000 | 93.9 (92.70 – 94.93) | 0.00 | 0.836 |
|  | 3 Years | 90.7 (86.48 – 93.65) | 0.00 | 0.590 | 90.5 (86.44 – 93.44) | 0.00 | 0.699 |
|  | > 5 Years | 89.4 (84.88 – 92.66) | 0.00 | 0.846 | 86.08 (71.60 – 93.81) | 74.37 | 0.002 |
| Graft Survival | 1 Year | 89.0 (85.07 – 91.93) | 0.00 | 0.851 | 89.2 (85.38 – 92.04) | 0.00 | 0.878 |
|  | 3 Years | 83.3 (72.95 – 90.18) | 54.64 | 0.051 | 80.0 (68.30 – 88.17) | 63.80 | 0.011 |
|  | > 5 Years | 69.2 (62.78 – 75.01) | 0.00 | 0.871 | 63.3 (48.65 – 75.82) | 64.55 | 0.023 |
| Graft Rejection | 1 Year | 28.6 (13.17 – 51.43) | 79.77 | 0.007 | 23.6 (10.51 – 44.75) | 77.59 | 0.004 |
|  | 3 Years | 55.5 (25.03 – 82.35) | 83.24 | 0.014 | 49.8 (31.61 – 68.02 | 66.74 | 0.050 |
|  | > 5 Years | 30.7 (20.70 – 43.02) | 0.00 | 0.480 | 37.1 (23.03 – 53.69) | 48.18 | 0.1223 |

##### Supplementary Table S5: Sensitivity analysis of primary and secondary outcomes according to location of studies

| Outcome Measure | Time Frame | United States | | | Europe | | |
| --- | --- | --- | --- | --- | --- | --- | --- |
|  |  | **Survival / Rate (95%CI)(%)** | **I^2^ (%)** | **P-Value** | **Survival / Rate (%)** | **I^2^ (%)** | **P-Value** |
| Patient Survival | 1 Year | 94.3 (93.17 – 95.32) | 0.00 | 0.945 | 94.4 (90.91 – 96.57) | 0.00 | 0.999 |
|  | 3 Years | 88.8 (85.96 – 91.08) | 56.42 | 0.006 | 91.8 (87.32 – 94.73) | 0.00 | 0.967 |
|  | > 5 Years | 80.9 (68.58 – 89.20) | 81.45 | 0.001 | 85.8 (76.51 – 91.77) | 48.86 | 0.048 |
| Graft Survival | 1 Year | 90.3 (88.72 – 91.58) | 0.50 | 0.441 | 92.2 (88.09 – 94.91) | 0.00 | 0.895 |
|  | 3 Years | 78.8 (71.68 – 84.51) | 88.46 | <0.0001 | 81.3 (75.48 – 85.98) | 0.00 | 0.809 |
|  | > 5 Years | 63.8 (53.30 – 73.05) | 73.45 | 0.010 | 74.9 (67.47 – 81.16) | 0.00 | 0.462 |
| Graft Rejection | 1 Year | 24.6 (15.90 – 36.00) | 92.57 | <0.0001 | 27.8 (17.35 – 41.49) | 75.55 | 0.001 |
|  | 3 Years | 31.1 (20.89 – 43.51) | 77.35 | 0.0001 | 40.3 (21.25 – 62.80) | 0.00 | N/A^1^ |
|  | > 5 Years | 36.2 (23.22 – 51.55) | 95.72 | <0.0001 | 40.2 (27.80 – 53.92) | 47.21 | 0.1084 |

1. Only one Europe study reported graft rejection in 3 years(3)

##### Supplementary Table S6: Univariate meta-regression of different factors on primary and secondary outcomes using random effects model.

| Outcome Measure | Time Frame | Moderator | Pooled Estimate | P-Value | Number of Studies Recruited |
| --- | --- | --- | --- | --- | --- |
| Patient Survival | 1 Year | Post-transplantation ATG Use | -0.33 | 0.04 | 16 |
|  |  | Pre-transplantation CD4 Count | 0.003 | <0.0001 | 16 |
|  |  | HIV-Associated Nephropathy (HIVAN) | -0.41 | 0.001 | 16 |
|  |  | Post-transplantation NNRTI Use | -0.44 | 0.015 | 13 |
|  |  | Avoiding Protease Inhibitors (PI Avoid) | 0.29 | <0.0001 | 15 |
|  |  | Post-transplantation PI Use | -0.39 | <0.0001 | 15 |
|  |  | Pre-transplantation PI Use | 0.47 | 0.015 | 15 |
|  | 3 Years | Induction Therapy Usage During Transplantation | -1.40 | <0.0001 | 12 |
|  |  | Pre-transplantation Viral Suppression | -6.16 | <0.0001 | 13 |
|  |  | Post-transplantation ATG Use | -1.06 | <0.0001 | 12 |
|  |  | Pre-transplantation CD4 Count | -0.003 | <0.0001 | 12 |
| Graft Survival | 1 Year | Hepatitis B Co-infection (HBV) | 6.17 | 0.01 | 13 |
|  |  | Hepatitis C Co-infection (HCV) | -0.68 | 0.04 | 19 |
|  |  | Pre-transplantation CD4 Count | -0.0006 | <0.0001 | 16 |
|  |  | Post-transplantation NNRTI Use | -1.53 | <0.0001 | 12 |
|  |  | Pre-transplantation NNRTI Use | -1.00 | <0.0001 | 13 |
|  |  | PI Avoid | 0.36 | 0.026 | 14 |
|  |  | Post-transplantation PI Use | -1.12 | 0.014 | 14 |
|  |  | Pre-transplantation PI Use | -0.61 | 0.019 | 15 |
|  |  | Viral Suppression After Transplantation | 1.19 | <0.0001 | 13 |
|  |  | Pre-transplantation Viral Suppression | 3.15 | <0.0001 | 17 |
|  | 3 Years | Hepatitis B Co-infection (HBV) | 3.64 | 0.016 | 12 |
|  |  | PI Avoid | 1.08 | <0.0001 | 11 |
|  |  | Post-transplantation PI Use | -2.17 | <0.0001 | 12 |
|  | 5 Years | Hepatitis C Co-infection (HCV) | -1.09 | 0.004 | 10 |
| Graft Rejection | 1 Year | HIV-Associated Nephropathy (HIVAN) | -1.07 | <0.0001 | 16 |
|  |  | Interleukin-2 (IL-2) Use | 0.87 | <0.0001 | 17 |
|  |  | Induction Therapy Usage | -0.63 | 0.042 | 17 |
|  |  | Post-transplantation NNRTI Use | 4.04 | <0.0001 | 13 |
|  |  | Pre-transplantation NNRTI Use | 0.91 | 0.014 | 12 |
|  |  | PI Avoid | -0.72 | <0.0001 | 12 |
|  |  | Post-transplantation PI Use | -0.21 | 0.023 | 12 |
|  |  | Viral Breakthrough | 0.96 | 0.007 | 11 |

#### **Supplementary Figures**

##### Supplementary Figure S1a-h: Baujat plots for heterogeneity assesssment

| Patient survival HR | 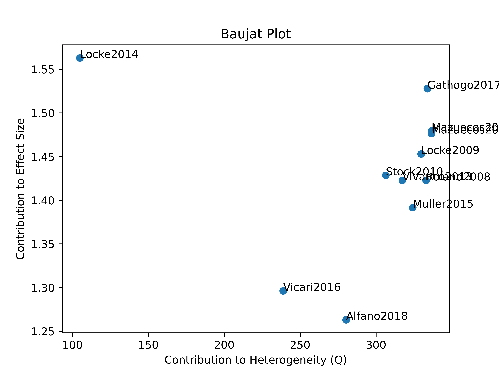 | 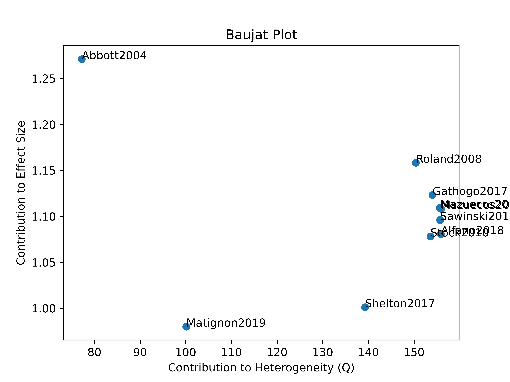 | 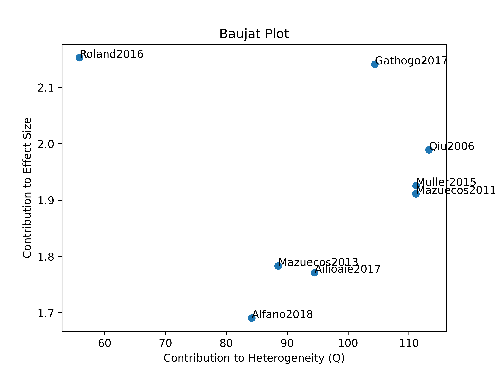 |
| --- | --- | --- | --- |
| Graft survival HR | **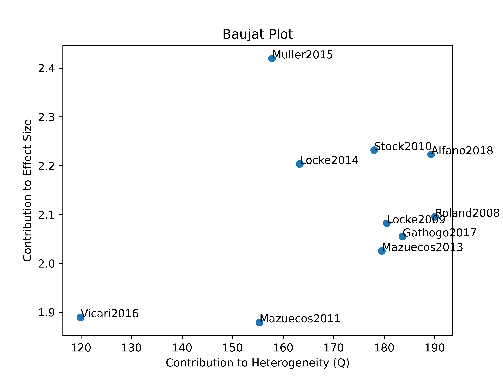** | 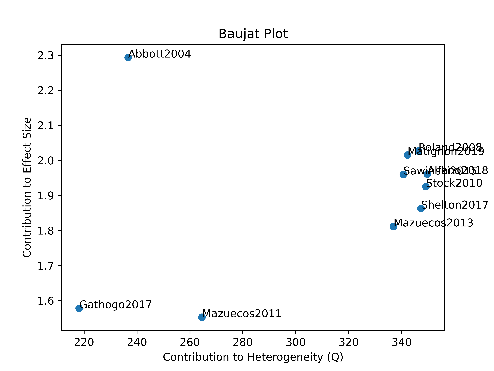 | 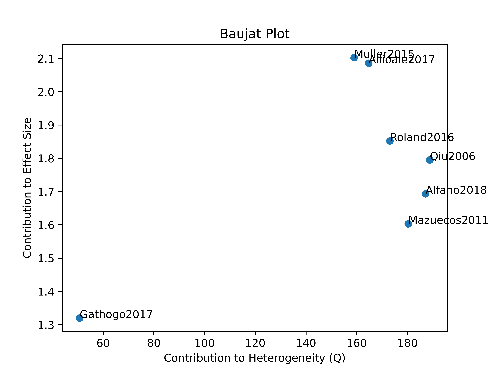 |
| Graft rejection HR | **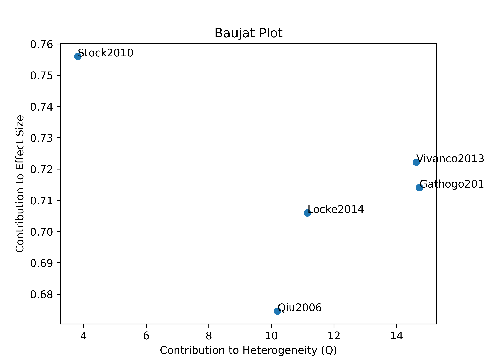** |  | 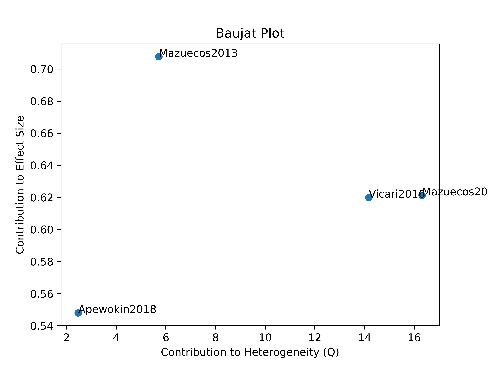 |
|  | 1 year | 3 years | > 5 years |

##### Supplementary Figure S2a. Result of quality assessments in traffic Light plot


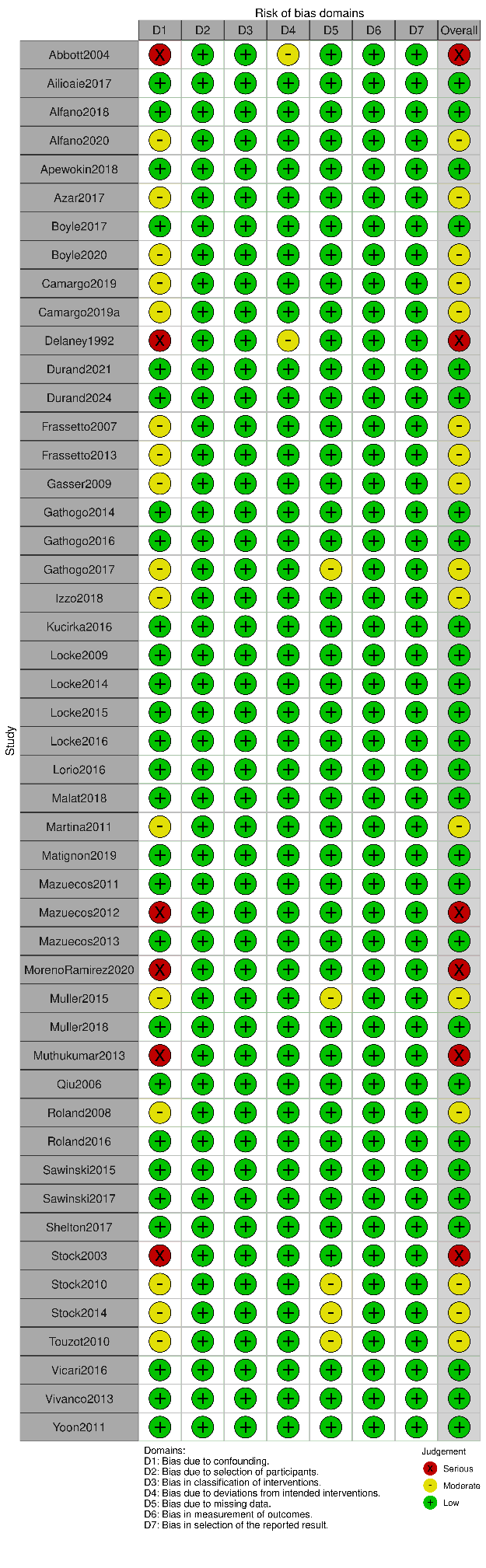


##### Supplementary Figure S2b. Result of quality assessments in summary plot


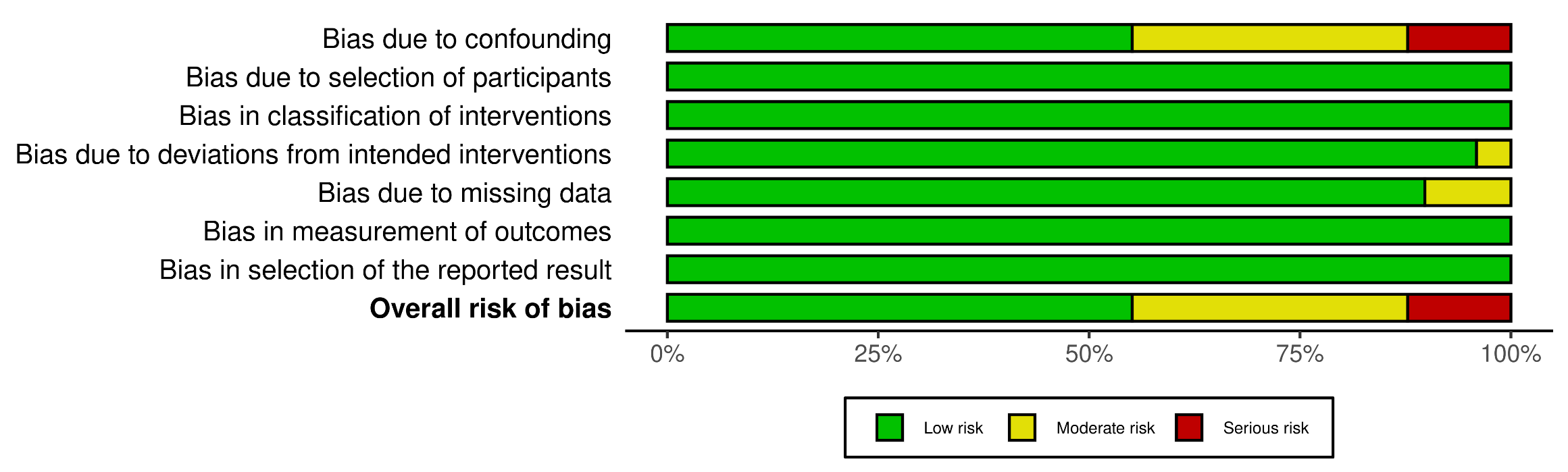


### Appendix 2. Search Strategy

**Embase via OVID < last searched on 2024 December 31> :**

1 HIV infection.mp. or exp Human immunodeficiency virus infection/ (854064)

2 HIV seropositivity.ab,ti. (1416)

3 acquired immune deficiency syndrome/ or Human immunodeficiency virus infection/ or HIV seropositive.mp. (412339)

4 HIV Infections/ or HIV Seropositivity/ or HIV seroposit*.mp. (164274)

5 kidney graft/ or Kidney Transplant*.mp. or transplantation/ or kidney transplantation/ (381825)

6 (renal transplant* or kidney transplant*).mp. [mp=title, abstract, heading word, drug trade name, original title, device manufacturer, drug manufacturer, device trade name, keyword heading word, floating subheading word, candidate term word] (207795)

7 anti retroviral therapy.mp. or exp antiretroviral therapy/ (75514)

8 (anti-retroviral therapy or antiretroviral therapy or Anti-retroviral therapy or Antiretroviral therapy or HAART or ART).mp. [mp=title, abstract, heading word, drug trade name, original title, device manufacturer, drug manufacturer, device trade name, keyword heading word, floating subheading word, candidate term word] (356205)

9 Anti-HIV Agents.mp. or exp anti human immunodeficiency virus agent/ (202934)

10 Graft Survival/ (80730)

11 transplant rejection.mp. or exp graft rejection/ (138328)

12 survival rate.mp. or exp survival rate/ (420103)

13 Treatment Outcome.mp. or outcome assessment/ or treatment outcome/ (1927546)

14 Viral Load.mp. or virus load/ (128364)

15 1 or 2 or 3 or 4 (850021)

16 5 or 6 (388665)

17 7 or 8 or 9 (510559)

18 10 or 11 or 12 or 13 or 14 (2540130)

19 15 and 16 and 17 and 18 (557)

20 Review.mp. or "systematic review"/ or "review"/ or "systematic review (topic)"/ (5157145)

21 19 not 20 (377)

22 Conference Abstract.mp. or conference abstract/ (2470804)

23 21 not 22 (354)

24 letter/ (1270663)

25 editorial/ (796385)

26 23 not (24 or 25) (322)

**MEDLINE via OVID <last searched on 2024 December 31>:**

Ovid MEDLINE(R) ALL

1 HIV infection.ab,ti. or HIV Infections/ (252365)

2 HIV seropositivity.mp. or exp HIV Seropositivity/ (24981)

3 exp Kidney Transplantation/ or kidney transplant.mp. (112445)

4 (renal transplant* or kidney transplant*).mp. [mp=title, book title, abstract, original title, name of substance word, subject heading word, floating sub-heading word, keyword heading word, organism supplementary concept word, protocol supplementary concept word, rare disease supplementary concept word, unique identifier, synonyms, population supplementary concept word, anatomy supplementary concept word] (126838)

5 anti retroviral therapy.mp. or Antiretroviral Therapy, Highly Active/ (24903)

6 exp Anti-HIV Agents/ae, pd, po, tu, to [Adverse Effects, Pharmacology, Poisoning, Therapeutic Use, Toxicity] (62454)

7 Graft Survival/ (53521)

8 transplant rejection.mp. or exp Graft Rejection/ (66900)

9 survival rate.mp. or Survival Rate/ (283159)

10 Viral Load.mp. or exp Viral Load/ (58316)

11 Treatment Outcome.mp. or Treatment Outcome/ (1188299)

12 7 or 8 or 9 or 10 or 11 (1547971)

13 5 or 6 (79127)

14 3 or 4 (126838)

15 1 or 2 (266768)

16 12 and 13 and 14 and 15 (84)

17 "Systematic Review"/ or "Review"/ or review.mp. (4103662)

18 16 not 17 (55)

**MEDLINE via PubMed <last searched on 2024 December 31>:**

(("hiv infections"[MeSH Terms] OR ("hiv"[All Fields] AND "infections"[All Fields]) OR "hiv infections"[All Fields] OR ("hiv"[All Fields] AND "infection"[All Fields]) OR "hiv infection"[All Fields]) AND ("kidney transplantation"[MeSH Terms] OR ("kidney"[All Fields] AND "transplantation"[All Fields]) OR "kidney transplantation"[All Fields] OR ("kidney"[All Fields] AND "transplant"[All Fields]) OR "kidney transplant"[All Fields]) AND ("adult"[MeSH Terms] OR "adult"[All Fields] OR "adults"[All Fields] OR "adult's"[All Fields])) NOT ("Case Reports"[Publication Type] OR ("renal dialysis"[MeSH Terms] OR ("renal"[All Fields] AND "dialysis"[All Fields]) OR "renal dialysis"[All Fields]) OR ("sars-cov-2"[MeSH Terms] OR "sars-cov-2"[All Fields] OR "covid"[All Fields] OR "covid-19"[MeSH Terms] OR "covid-19"[All Fields])) (354)

**CENTRAL via Cochrane Library <last searched on 2024 December 31>:**

1 MeSH descriptor: [HIV] explode all trees

2 MeSH descriptor: [Acquired Immunodeficiency Syndrome] explode all trees

3 MeSH descriptor: [Kidney Failure, Chronic] explode all trees

4 MeSH descriptor: [Kidney Failure, Chronic] explode all trees

5 MeSH descriptor: [Kidney Transplantation] explode all trees

6 #1 OR #2

7 #3 OR #4 OR #5

8 #6 AND #7

**The search strategy was refined based on valuable comments and evaluations provided by Dr. Wendy Kwok and Dr. Jonathan Ciofani.**

### Appendix 3. Eligibility criteria

| Criteria | Inclusion | Exclusion |
| --- | --- | --- |
| **Participants(P)** | Adult PWH who have undergone kidney transplantation. | Studies involving PWH who have not undergone kidney transplantation only. |
| **Interventions(I)** | Kidney transplantation in PWH, including pre-transplant preparation and post-transplant care. | Interventions unrelated to kidney transplantation in PWH. |
| **Comparators(C)** | Individuals with ESKD who are not living with HIV/AIDS and have undergone kidney transplantation. | Non-ESKD individuals or transplants unrelated to kidney. |
| **Outcomes(O)** | Primary:   - Graft survival,   Secondary:   - Graft rejection, - Opportunistic infections, - Severe infections requiring hospitalizations, - Patient survival - Impact of ART and immunosuppressants - HIV viral load, - Reactivation of HBV/HCV | Outcomes not related to graft and patient survival, rejection, or post-transplant complications. |
| **Study Design** | - Randomized controlled trials, - Prospective or retrospective observational studies, - Registry data analyses | - Any trials involving duplicated data - Animal studies, - Systematic reviews, - Literature reviews, - Editorials, letters, opinion pieces, and commentaries, - Conference abstracts, - Case reports, - Case series reporting less than 10 patients |
| **Time Frame** | Studies reporting outcome in at least 12 months of follow-up. | Studies with incomplete follow-up data or less than 12 months follow-up. |
| **Report Characteristics - Years** | Studies published between 1990 and 2024. | Studies published before 1990. |
| **Report Characteristics - Language** | Studies published in English. | Studies not published in English. |

### Appendix 4. References

1. Abbott KC, Swanson SJ, Agodoa LYC, Kimmel PL. Human Immunodeficiency Virus Infection and Kidney Transplantation in the Era of Highly Active Antiretroviral Therapy and Modern Immunosuppression. *Journal of the American Society of Nephrology*. 2004;15(6): 1633–1639. https://doi.org/10.1097/01.ASN.0000127987.19470.3A.

2. Ailioaie O, Arzouk N, Valantin M, Tourret J, Calin R, Turinici M, et al. Infectious complications in HIV-infected kidney transplant recipients. *International Journal of STD & AIDS*. 2018;29(4): 341–349. https://doi.org/10.1177/0956462417726213.

3. Alfano G, Mori G, Fontana F, Dolci G, Baisi A, Ligabue G, et al. Clinical outcome of kidney transplantation in HIV-infected recipients: a retrospective study. *International Journal of STD & AIDS*. 2018;29(13): 1305–1315. https://doi.org/10.1177/0956462418779659.

4. Alfano G, Guaraldi G, Fontana F, Franceschini E, Dolci G, Mussini C, et al. Role of Maraviroc in minimizing the risk of graft rejection in HIV‐infected kidney transplant recipients. *Transplant Infectious Disease*. 2020;22(4): e13294. https://doi.org/10.1111/tid.13294.

5. Apewokin S, Madan R, Restrepo A, Hemmige VS, Arora S. Clinical and Health Care Utilization Outcomes of Kidney Transplantation in HIV-Positive Recipients: A Nationwide Analysis From 2008-2013. *Transplantation Proceedings*. 2018;50(10): 3361–3366. https://doi.org/10.1016/j.transproceed.2018.08.002.

6. Azar MM, Malinis MF, Moss J, Formica RN, Villanueva MS. Integrase strand transferase inhibitors: the preferred antiretroviral regimen in HIV-positive renal transplantation. *International Journal of STD & AIDS*. 2017;28(5): 447–458. https://doi.org/10.1177/0956462416651528.

7. Boyle SM, Malat G, Harhay MN, Lee DH, Pang L, Talluri S, et al. Association of tenofovir disoproxil fumarate with primary allograft survival in HIV ‐positive kidney transplant recipients. *Transplant Infectious Disease*. 2017;19(4): e12727. https://doi.org/10.1111/tid.12727.

8. Boyle SM, Fehr K, Deering C, Raza A, Harhay MN, Malat G, et al. Barriers to kidney transplant evaluation in HIV‐positive patients with advanced kidney disease: A single‐center study. *Transplant Infectious Disease*. 2020;22(2): e13253. https://doi.org/10.1111/tid.13253.

9. Camargo JF, Pallikkuth S, Moroz I, Natori Y, Alcaide ML, Rodriguez A, et al. Pretransplant Levels of C-Reactive Protein, Soluble TNF Receptor-1, and CD38+HLADR+ CD8 T Cells Predict Risk of Allograft Rejection in HIV+ Kidney Transplant Recipients. *Kidney International Reports*. 2019;4(12): 1705–1716. https://doi.org/10.1016/j.ekir.2019.08.006.

10. Camargo JF, Anjan S, Chin-Beckford N, Morris MI, Abbo LM, Simkins J, et al. Clinical outcomes in HIV+/HCV+ coinfected kidney transplant recipients in the pre- and post-direct-acting antiviral therapy eras: 10-Year single center experience. *Clinical Transplantation*. 2019;33(5): e13532. https://doi.org/10.1111/ctr.13532.

11. Delaney V, Sumrani VN, Hong J, Davis R, Sommer B. The course of HIV disease in renal allograft recipients. *Transplant International*. 1992;5: S129–S132. https://doi.org/10.1111/tri.1992.5.s1.129.

12. Durand CM, Zhang W, Brown DM, Yu S, Desai N, Redd AD, et al. A prospective multicenter pilot study of HIV-positive deceased donor to HIV-positive recipient kidney transplantation: HOPE in action. *American Journal of Transplantation*. 2021;21(5): 1754–1764. https://doi.org/10.1111/ajt.16205.

13. *Safety of Kidney Transplantation from Donors with HIV | New England Journal of Medicine*. https://www.nejm.org/doi/full/10.1056/NEJMoa2403733 [Accessed 26th December 2024].

14. Frassetto LA, Browne M, Cheng A, Wolfe AR, Roland ME, Stock PG, et al. Immunosuppressant Pharmacokinetics and Dosing Modifications in HIV-1 Infected Liver and Kidney Transplant Recipients. *American Journal of Transplantation*. 2007;7(12): 2816–2820. https://doi.org/10.1111/j.1600-6143.2007.02007.x.

15. Frassetto L, Floren L, Barin B, Browne M, Wolfe A, Roland M, et al. Changes in clearance, volume and bioavailability of immunosuppressants when given with HAART in HIV‐1 infected liver and kidney transplant recipients. *Biopharmaceutics & Drug Disposition*. 2013;34(8): 442–451. https://doi.org/10.1002/bdd.1860.

16. Gasser O, Bihl F, Sanghavi S, Rinaldo C, Rowe D, Hess C, et al. Treatment-dependent Loss of Polyfunctional CD8+ T-cell Responses in HIV-infected Kidney Transplant Recipients Is Associated with Herpesvirus Reactivation. *American Journal of Transplantation*. 2009;9(4): 794–803. https://doi.org/10.1111/j.1600-6143.2008.02539.x.

17. Gathogo EN, Hamzah L, Hilton R, Marshall N, Ashley C, Harber M, et al. Kidney transplantation in HIV-positive adults: the UK experience. *International Journal of STD & AIDS*. 2014;25(1): 57–66. https://doi.org/10.1177/0956462413493266.

18. Gathogo E, Harber M, Bhagani S, Levy J, Jones R, Hilton R, et al. Impact of Tacrolimus Compared With Cyclosporin on the Incidence of Acute Allograft Rejection in Human Immunodeficiency Virus–Positive Kidney Transplant Recipients. *Transplantation*. 2016;100(4): 871–878. https://doi.org/10.1097/TP.0000000000000879.

19. Gathogo EN, Shah S, Post FA. Kidney transplant outcomes in HIV serodiscordant recipient pairs. *AIDS*. 2017;31(8): 1199–1201. https://doi.org/10.1097/QAD.0000000000001457.

20. Izzo I, Casari S, Bossini N, Forleo MA, Sandrini S, Focà E, et al. Effectiveness of kidney transplantation in HIV-infected recipients under combination antiretroviral therapy: a single-cohort experience (Brescia, Northern Italy). *Infection*. 2018;46(1): 77–82. https://doi.org/10.1007/s15010-017-1092-2.

21. Kucirka LM, Durand CM, Bae S, Avery RK, Locke JE, Orandi BJ, et al. Induction Immunosuppression and Clinical Outcomes in Kidney Transplant Recipients Infected With Human Immunodeficiency Virus. *American Journal of Transplantation*. 2016;16(8): 2368–2376. https://doi.org/10.1111/ajt.13840.

22. Locke JE. Renal Transplant in HIV-Positive Patients: Long-term Outcomes and Risk Factors for Graft Loss. *Archives of Surgery*. 2009;144(1): 83. https://doi.org/10.1001/archsurg.2008.508.

23. Locke JE, James NT, Mannon RB, Mehta SG, Pappas PG, Baddley JW, et al. Immunosuppression Regimen and the Risk of Acute Rejection in HIV-Infected Kidney Transplant Recipients. *Transplantation*. 2014;97(4): 446–450. https://doi.org/10.1097/01.TP.0000436905.54640.8c.

24. Locke JE, Reed RD, Mehta SG, Durand C, Mannon RB, MacLennan P, et al. Center-Level Experience and Kidney Transplant Outcomes in HIV-Infected Recipients. *American Journal of Transplantation*. 2015;15(8): 2096–2104. https://doi.org/10.1111/ajt.13220.

25. Locke JE, Shelton BA, Reed RD, MacLennan PA, Mehta S, Sawinski D, et al. Identification of Optimal Donor–Recipient Combinations Among Human Immunodeficiency Virus (HIV)–Positive Kidney Transplant Recipients. *American Journal of Transplantation*. 2016;16(8): 2377–2383. https://doi.org/10.1111/ajt.13847.

26. Lorio MA, Rosa R, Suarez JF, Ruiz P, Ciancio G, Burke GW, et al. Influence of immune activation on the risk of allograft rejection in human immunodeficiency virus-infected kidney transplant recipients. *Transplant Immunology*. 2016;38: 40–43. https://doi.org/10.1016/j.trim.2016.06.001.

27. Malat GE, Boyle SM, Jindal RM, Guy S, Xiao G, Harhay MN, et al. Kidney Transplantation in HIV-Positive Patients: A Single-Center, 16-Year Experience. *American Journal of Kidney Diseases*. 2019;73(1): 112–118. https://doi.org/10.1053/j.ajkd.2018.02.352.

28. Martina MN, Cofan F, Suarez A, Masso E, Trullas JC, Cervera C, et al. Kidney Transplantation and Waiting List for Renal Transplantation for Human Immunodeficiency Virus Patients. *Transplantation Proceedings*. 2011;43(6): 2179–2181. https://doi.org/10.1016/j.transproceed.2011.05.024.

29. Matignon M, Lelièvre J, Lahiani A, Abbassi K, Desvaux D, Diallo A, et al. Low incidence of acute rejection within 6 months of kidney transplantation in HIV‐infected recipients treated with raltegravir: the Agence Nationale de Recherche sur le Sida et les Hépatites Virales (ANRS) 153 TREVE trial. *HIV Medicine*. 2019;20(3): 202–213. https://doi.org/10.1111/hiv.12700.

30. Mazuecos A, Fernandez A, Andres A, Gomez E, Zarraga S, Burgos D, et al. HIV infection and renal transplantation. *Nephrology Dialysis Transplantation*. 2011;26(4): 1401–1407. https://doi.org/10.1093/ndt/gfq592.

31. Mazuecos A, Rodriguez Benot A, Moreno A, Burgos D, Aguera M, Garcia Alvarez T, et al. Renal Replacement Therapy in Patients With HIV Infection in a European Region: Outcomes Following Renal Transplantation. *Transplantation Proceedings*. 2012;44(7): 2053–2056. https://doi.org/10.1016/j.transproceed.2012.07.082.

32. Mazuecos A, Fernandez A, Zarraga S, Andres A, Rodriguez-Benot A, Jimenez C, et al. High incidence of delayed graft function in HIV-infected kidney transplant recipients. *Transplant International*. 2013;26(9): 893–902. https://doi.org/10.1111/tri.12147.

33. Moreno-Ramirez M, Villanego F, Vigara LA, Cazorla JM, Naranjo J, Garcia T, et al. Direct-Acting Antiretroviral Therapy in Renal Transplant Recipients With Human Immunodeficiency Virus–Hepatitis C Virus Coinfection: Report of Our Experience and Literature Review. *Transplantation Proceedings*. 2020;52(2): 523–526. https://doi.org/10.1016/j.transproceed.2019.12.009.

34. Muller E. Transplantation in resource-limited setting: using HIV-positive donors for HIV-positive patients. *Clinical Nephrology*. 2015;83 (2015)(S1): 39–41. https://doi.org/10.5414/CNP83S0039.

35. Muller E, Barday Z. HIV-Positive Kidney Donor Selection for HIV-Positive Transplant Recipients. *Journal of the American Society of Nephrology*. 2018;29(4): 1090–1095. https://doi.org/10.1681/ASN.2017080853.

36. Muthukumar T, Afaneh C, Ding R, Tsapepas D, Lubetzky M, Jacobs S, et al. HIV-Infected Kidney Graft Recipients Managed With an Early Corticosteroid Withdrawal Protocol: Clinical Outcomes and Messenger RNA Profiles. *Transplantation*. 2013;95(5): 711–720. https://doi.org/10.1097/TP.0b013e31827ac322.

37. Qiu J, Terasaki PI, Waki K, Cai J, Gjertson DW. HIV-Positive Renal Recipients Can Achieve Survival Rates Similar to Those of HIV-Negative Patients. *Transplantation*. 2006;81(12): 1658–1661. https://doi.org/10.1097/01.tp.0000226074.97314.e0.

38. Roland ME, Barin B, Carlson L, Frassetto LA, Terrault NA, Hirose R, et al. HIV-Infected Liver and Kidney Transplant Recipients: 1- and 3-Year Outcomes. *American Journal of Transplantation*. 2008;8(2): 355–365. https://doi.org/10.1111/j.1600-6143.2007.02061.x.

39. Roland ME, Barin B, Huprikar S, Murphy B, Hanto DW, Blumberg E, et al. Survival in HIV-positive transplant recipients compared with transplant candidates and with HIV-negative controls: *AIDS*. 2015; 1. https://doi.org/10.1097/QAD.0000000000000934.

40. Sawinski D, Forde KA, Eddinger K, Troxel AB, Blumberg E, Tebas P, et al. Superior outcomes in HIV-positive kidney transplant patients compared with HCV-infected or HIV/HCV-coinfected recipients. *Kidney International*. 2015;88(2): 341–349. https://doi.org/10.1038/ki.2015.74.

41. Sawinski D, Shelton BA, Mehta S, Reed RD, MacLennan PA, Gustafson S, et al. Impact of Protease Inhibitor–Based Anti-Retroviral Therapy on Outcomes for HIV+ Kidney Transplant Recipients. *American Journal of Transplantation*. 2017;17(12): 3114–3122. https://doi.org/10.1111/ajt.14419.

42. Shelton BA, Mehta S, Sawinski D, Reed RD, MacLennan PA, Gustafson S, et al. Increased Mortality and Graft Loss With Kidney Retransplantation Among Human Immunodeficiency Virus (HIV)–Infected Recipients. *American Journal of Transplantation*. 2017;17(1): 173–179. https://doi.org/10.1111/ajt.13922.

43. Stock PG, Roland ME, Carlson L, Freise CE, Roberts JP, Hirose R, et al. Kidney and liver transplantation in human immunodeficiency virus-infected patients: a pilot safety and efficacy study. *Transplantation*. 2003;76(2): 370–375. https://doi.org/10.1097/01.TP.0000075973.73064.A6.

44. Stock PG, Barin B, Murphy B, Hanto D, Diego JM, Light J, et al. Outcomes of Kidney Transplantation in HIV-Infected Recipients. *New England Journal of Medicine*. 2010;363(21): 2004–2014. https://doi.org/10.1056/NEJMoa1001197.

45. Stock PG, Barin B, Hatano H, Rogers RL, Roland ME, Lee TH, et al. Reduction of HIV persistence following transplantation in HIV-infected kidney transplant recipients. *American Journal of Transplantation: Official Journal of the American Society of Transplantation and the American Society of Transplant Surgeons*. 2014;14(5): 1136–1141. https://doi.org/10.1111/ajt.12699.

46. Touzot M, Pillebout E, Matignon M, Tricot L, Viard JP, Rondeau E, et al. Renal Transplantation in HIV‐Infected Patients: The Paris Experience. *American Journal of Transplantation*. 2010;10(10): 2263–2269. https://doi.org/10.1111/j.1600-6143.2010.03258.x.

47. Vicari AR, Spuldaro F, Sandes‐Freitas TV, Cristelli MP, Requião‐Moura LR, Reusing JO, et al. Renal transplantation in human immunodeficiency virus‐infected recipients: a case–control study from the Brazilian experience. *Transplant Infectious Disease*. 2016;18(5): 730–740. https://doi.org/10.1111/tid.12592.

48. Vivanco M, Friedmann P, Xia Y, Klair T, Marfo K, De Boccardo G, et al. Campath induction in HCV and HCV/HIV-seropositive kidney transplant recipients. *Transplant International*. 2013;26(10): 1016–1026. https://doi.org/10.1111/tri.12167.

49. Yoon SC, Hurst FP, Jindal RM, George SA, Neff RT, Agodoa LY, et al. Trends in Renal Transplantation in Patients With Human Immunodeficiency Virus Infection: An Analysis of the United States Renal Data System. *Transplantation*. 2011;91(8): 864–868. https://doi.org/10.1097/TP.0b013e31820f081f.
